# Daughter-Specific Transcription Factors Regulate Cell Size Control in Budding Yeast

**DOI:** 10.1371/journal.pbio.1000221

**Published:** 2009-10-20

**Authors:** Stefano Di Talia, Hongyin Wang, Jan M. Skotheim, Adam P. Rosebrock, Bruce Futcher, Frederick R. Cross

**Affiliations:** 1 The Rockefeller University, New York, New York, United States of America; 2 Department of Molecular Genetics and Microbiology, SUNY at Stony Brook, Stony Brook, New York, United States of America; University of California Santa Cruz, United States of America

## Abstract

In budding yeast, asymmetric cell division yields a larger mother and a smaller daughter cell, which transcribe different genes due to the daughter-specific transcription factors Ace2 and Ash1. Cell size control at the Start checkpoint has long been considered to be a main regulator of the length of the G1 phase of the cell cycle, resulting in longer G1 in the smaller daughter cells. Our recent data confirmed this concept using quantitative time-lapse microscopy. However, it has been proposed that daughter-specific, Ace2-dependent repression of expression of the G1 cyclin *CLN3* had a dominant role in delaying daughters in G1. We wanted to reconcile these two divergent perspectives on the origin of long daughter G1 times. We quantified size control using single-cell time-lapse imaging of fluorescently labeled budding yeast, in the presence or absence of the daughter-specific transcriptional regulators Ace2 and Ash1. Ace2 and Ash1 are not required for efficient size control, but they shift the domain of efficient size control to larger cell size, thus increasing cell size requirement for Start in daughters. Microarray and chromatin immunoprecipitation experiments show that Ace2 and Ash1 are direct transcriptional regulators of the G1 cyclin gene *CLN3*. Quantification of cell size control in cells expressing titrated levels of Cln3 from ectopic promoters, and from cells with mutated Ace2 and Ash1 sites in the *CLN3* promoter, showed that regulation of *CLN3* expression by Ace2 and Ash1 can account for the differential regulation of Start in response to cell size in mothers and daughters. We show how daughter-specific transcriptional programs can interact with intrinsic cell size control to differentially regulate Start in mother and daughter cells. This work demonstrates mechanistically how asymmetric localization of cell fate determinants results in cell-type-specific regulation of the cell cycle.

## Abstract 2

The asymmetric localization of cell fate determinants results in asymmetric cell cycle control in budding yeast.

## Author Summary

Asymmetric cell division is a universal mechanism for generating differentiated cells. The progeny of such divisions can often display differential cell cycle regulation. This study addresses how differential regulation of gene expression in the progeny of a single division can alter cell cycle control. In budding yeast, asymmetric cell division yields a bigger ‘mother’ cell and a smaller ‘daughter’ cell. Regulation of gene expression is also asymmetric because two transcription factors, Ace2 and Ash1, are specifically localized to the daughter. Cell size has long been proposed as important for the regulation of the cell cycle in yeast. Our work shows that Ace2 and Ash1 regulate size control in daughter cells: daughters ‘interpret’ their size as smaller, making size control more stringent and delaying cell cycle commitment relative to mother cells of the same size. This asymmetric interpretation of cell size is associated with differential regulation of the G1 cyclin *CLN3* by Ace2 and Ash1, at least in part via direct binding of these factors to the *CLN3* promoter. *CLN3* is the most upstream regulator of Start, the initiation point of the yeast cell cycle, and differential regulation of *CLN3* accounts for most or all asymmetric regulation of Start in budding yeast mother and daughter cells.

## Introduction

At the Start transition in G1, budding yeast cells integrate internal and external cues into an all-or-none commitment to a new round of cell division [1],[2]. Cell division is asymmetric, producing a smaller daughter cell and a larger mother cell [3]. Mother cells progress through Start more quickly than daughter cells [3],[4]. The regulation of G1 phase is composed of two independent modules separated by the nuclear exit of the transcriptional repressor Whi5 [5]: a cell size sensing module, which extends G1 in small cells to allow additional growth before Start [5], and a subsequent size-independent module [5],[6]. The fast and coherent transition between the two modules likely coincides with commitment to the cell cycle and is driven by transcriptional positive feedback [7]. The G1 cyclin Cln3 is the most upstream activator of the Start transition [8],[9],[10],[11],[12] and the main regulator of the size-sensing module. Cln3 initiates inactivation of Whi5 [13],[14] and expression of SBF/MBF dependent genes, including the G1 cyclins *CLN1* and *CLN2* [9],[11],[12],[15],[16]. Subsequent positive feedback of Cln1 and Cln2 on SBF/MBF dependent transcription ensures fast and coherent commitment to the cell cycle [7].

Cell size control is thought to regulate the length of the G1 phase of the cell cycle [4],[5],[17],[18]. In budding yeast, cell size control is readily detectable in daughter cells but much less obvious in mother cells. In part this is because mother cells are almost always born larger than daughters [3], but it has also been shown that daughters are slower to pass Start than mothers even when both are made equally large (greater than normal mother or daughter size) [19]. This finding suggested some asymmetry in Start control between mothers and daughters beyond that due to different cell size; differential gene expression in mothers and daughters could provide such asymmetry.

Regulation of gene expression is asymmetric in mother and daughter cells as a result of the daughter-specific localization of the transcription factors Ace2 and Ash1. Ace2 enters mother and daughter nuclei during mitotic exit [20],[21]. Asymmetric localization of Ace2 is due to the Mob2-Cbk1 complex [20],[21],[22], which promotes nuclear retention of Ace2 specifically in the newborn daughter nucleus, leading to daughter-specific expression of a number of genes [20],[21],[22],[23],[24]. Daughter-specific localization of Ash1 is achieved through active transport of *ASH1* mRNA to the bud tip and consequent preferential accumulation of Ash1 in the daughter nucleus [25]. Ash1 represses expression of the *HO* endonuclease gene responsible for mating type switching [26],[27], thus restricting *HO* expression to mother cells.

Recently, Ace2 was shown to cause a daughter-specific G1 delay, acting indirectly through “Daughter Delay Elements (DDE)” 5′ to the *CLN3* coding sequence to reduce *CLN3* expression in daughters [28]. In that work, it was proposed that this Ace2-dependent delay is the only reason that daughters have a longer G1 than mothers. Cell size was proposed to play no role in controlling the length of G1 [28]. This proposal is incompatible with our recent finding that small cells display very efficient size control, requiring a significantly longer period of growth to attain a sufficient size before exiting G1 [5]. Here, we resolve this conflict and further investigate the differences between mother and daughter cell cycle control by analyzing the interaction between daughter-specific transcriptional programs, cell size control, and irreversible commitment to the cell cycle at Start.

## Results

### Differential Regulation of Start in Mothers and Daughters Is Dependent on Ace2 and Ash1

**Figure 1:**
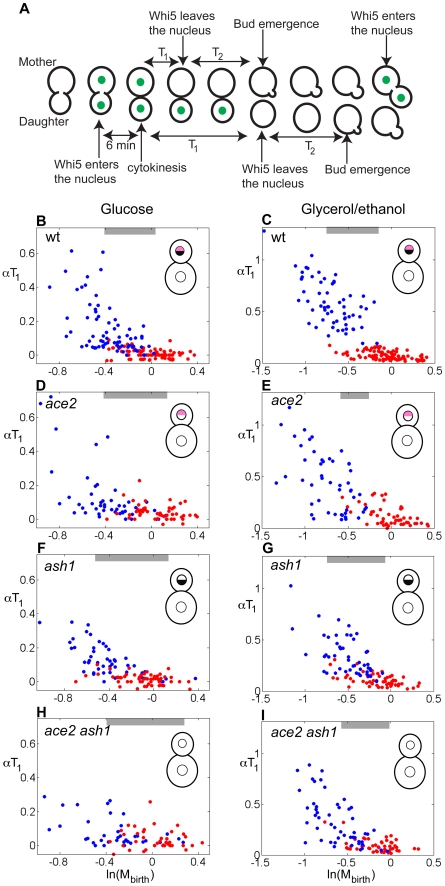
Differential regulation of Start is dependent on Ace2 and Ash1. (A) Illustration of the separation of G1 into two intervals, T_1_ and T_2_, by using Whi5-GFP. The total duration of G1 is T_1_+T_2_. (B–H) Correlation between αT_1_ and ln(M_birth_) for cells grown in glucose or glycerol/ethanol. (B–C) wild-type, (D–E) *ace2*, (F–G) *ash1*, (H–I) *ace2 ash1*. Red dots, mothers; blue dots, daughters. Inset: cartoon illustrating presence of Ace2 or Ash1 in mother and daughter nuclei; black semicircle, Ace2; pink semicircle, Ash1. Gray bars indicate the region of size overlap used for the analysis presented in Table 1.

G1 (defined operationally as the unbudded period of the cell cycle) can be decomposed into two independent steps, of duration T_1_ and T_2_, respectively, separated by exit from the nucleus of the transcriptional repressor Whi5 (Figure 1A) [5]. We previously used time-lapse fluorescence microscopy of yeast expressing *WHI5-GFP* and *ACT1pr-DsRed* [5] to simultaneously measure the duration of T_1_, measured by the interval of Whi5 nuclear residence, and cell size, measured using total cell fluorescence expressed from the constitutive *ACT1pr-DsRed* [5]. T_2_, the time between Whi5 nuclear exit and budding, is similar in mothers and daughters and is largely independent of cell size [5],[6]. T_1_ is extremely short in mothers but of significant duration in daughters [5],[6]. G1 size control is readily detected in small daughter cells, and maps specifically to the T_1_ interval [5].

Smaller cells have a longer T_1_, allowing growth to a larger size before cell cycle entry. This links birth size to T_1_ duration. Given exponential growth of single cells [5],[29], the size at Whi5 exit, *M*_1_, is related to the size at birth, *M*_birth_, through the period T_1_ by the simple formula: *M*_1_ = *M*_birth_e^α*T*1^, where α is the growth rate for exponential growth. This expression yields: αT_1_ = ln(*M*_1_)–ln(*M*_birth_). The correlation between α*T*_1_ and ln(*M*_birth_) characterizes the efficiency of size control. If there is efficient size control, then T_1_ should become larger as ln(*M*_birth_) becomes smaller, because cells born smaller require a longer period of growth to promote Start. Specifically, the slope of the linear fit of the plot of α*T*_1_ against ln(*M*_birth_) should be −1 in the case of perfect size control (that is, an exact size at which Start is invariably executed) and 0 in the absence of size control [5],[30].

The different duration of the period T_1_ in mothers and daughters could in principle be solely a consequence of size control imposing a delay in the smaller daughter cells [3]. We analyzed the correlation between αT_1_ and ln(M_birth_), comparing mothers and daughters binned for very similar size at birth (binning was necessary to ensure sufficient numbers of cells of a given size for statistical comparisons). This comparison demonstrates an increase in αT_1_ in daughters compared to mothers of similar size (Figure 1B, 1C; regions marked with bars) (*p* values<10^−6^; Table S4). This delay in Start is most readily detectable in glycerol-ethanol medium (Figure 1C) (*p* value<10^−70^; Table S4), in which cell growth is much slower than in glucose medium. Slower growth means that the mother cell feeds less biomass into the daughter cell, resulting in smaller daughter size at the time of cell division [3]. The resulting population of very small daughters enhances detection of size control (Figure 1C) [5]. In glycerol-ethanol, across the domain of size overlap in mother-daughter size at birth, daughters exhibit clear size control (slope ∼−0.8) while mothers exhibit essentially none (slope ∼0). This increase in αT_1_ in daughters with respect to mothers of equal size is consistent with previous findings of a daughter-specific delay, above and beyond the delay needed to achieve equivalent size [19],[28].

Laabs and collaborators had previously implicated the daughter-specific transcription factor Ace2 in delayed exit from G1 in daughters [28]. Ash1 is a second daughter-specific transcription factor [26],[27], and Ace2 contributes to the expression of *ASH1* in daughter cells [31]. Ash1 might therefore be the effector of the Ace2-induced daughter delay, or it could independently contribute to daughter delay. We analyzed the correlation between αT_1_ and ln(M_birth_) in *ace2* and *ash1* single and double mutants (for a complete list of strains and plasmids used in this study, see Table S1 and Table S2).

*ace2 ash1* mothers and daughters that were born at similar sizes exhibited similar αT_1_ values, failing to display the daughter-specific delay seen in wild-type (Figure 1H, 1I, and Table 1). Furthermore, only very small *ace2 ash1* daughters from glycerol/ethanol cultures displayed efficient size control. It is important to note that the mutant still displayed efficient size control by our metric; the effect of the deletions was to shift the size domain where efficient size control could be detected, not to eliminate size control per se.

**Table 1:** Average daughter delay in newborn cells of the same size.

	Wild-Type	*ace2*	*ash1*	*ace2 ash1*
Daughter-mother delay in glucose	8±1 min	2±3 min (0.06)	6±1 min (0.15)	3±2 min (0.03)
Daughter-mother delay in gly/eth	87±9 min	16±13 min (<10^−5^)	40±8 min (<10^−4^)	17±9 min (<10^−7^)
	**Wild-Type**	ACE2*	ASH1*	ACE2* ASH1*
Daughter-mother delay in glucose	8±1 min	1.3±0.9 min (<10^−5^)	5±1 min (0.03)	1.3±0.9 min (10^−5^)
Daughter-mother delay in gly/eth	87±9 min	37±12 min (<10^−3^)	19±7 min (10^−8^)	5±7 min (<10^−12^)
	**Wild-Type**	**Ace2/Swi5 Sites Mutated**	**Ash1 sites Mutated**	**Ace2/Swi5 and Ash1 Sites Mutated**
Daughter-mother delay in glucose	8±1 min	8±2 min (0.82)	10±2 min (0.37)	7±1 min (0.48)
Daughter-mother delay in gly/eth	87±9 min	46±7 min (<10^−3^)	47±15 min (0.02)	54±9 min (0.008)
	**Wild-Type**	***cln3***	***ADH1pr-CLN3***	***nxCDC28pr-CLN3***
Daughter-mother delay in glucose	8±1 min	3±1 min (<10^−3^)	N/A	3±1 min (<10^−3^)
Daughter-mother delay in gly/eth	87±9 min	9±13 min (<10^−6^)	22±10 min (<10^−5^)	33±12 min (<10^−3^)

**Figure 2:**
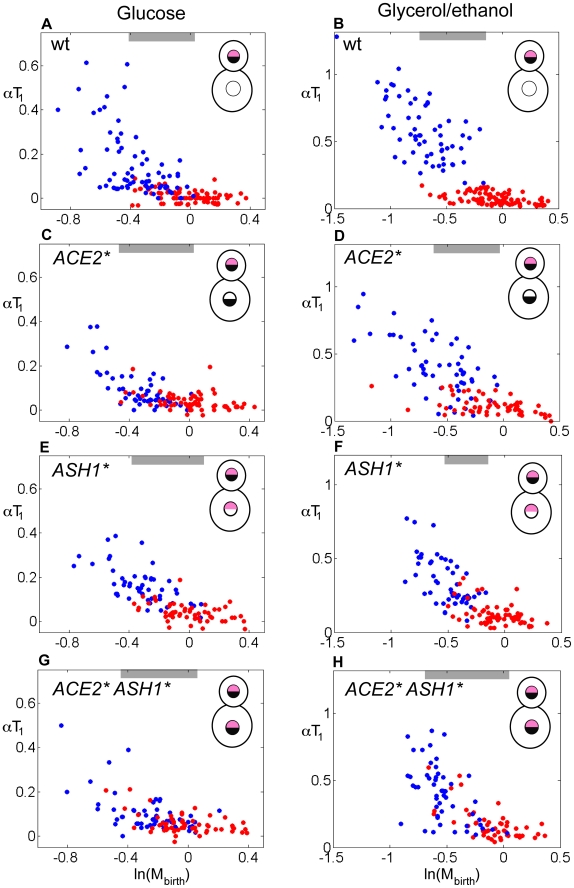
Symmetric localization of Ace2 and Ash1 result in symmetric control of Start in mothers and daughters. (A–H) Correlation between αT_1_ and ln(M_birth_) for cells grown in glucose or glycerol/ethanol. (A–B) wild-type, (C–D) ACE2*, (E–F) ASH1*, (G–H) ACE2* ASH1*. Red dots, mothers; blue dots, daughters. Black semicircle, Ace2; pink semicircle, Ash1. Asterisks indicate the dominant mutant forms. Gray bars indicate the region of size overlap used for the analysis presented in Table 1.

The region of overlap in size at birth of mothers and daughters was evaluated for every genotype separately (see gray bars in Figures 1, 2, 6, and 7). Data for the duration of T_1_ in this region were divided in small bins and the daughter delays (i.e., average excess in T_1_ for daughters over mothers) were computed for every size bin with representation of both mothers and daughters. The results were averaged across all these size bins. This definition of daughter delay is largely independent of the uneven distribution of cell size at birth in the region of overlap. The *p* value, computed by *t* test, for the hypothesis that the mutant daughter delay is the same as the wild-type daughter delay is indicated in parentheses. The statistical significance of difference in T_1_ times between mothers and daughters in the region of size overlap is presented in Table S4. Asterisks indicate the dominant mutant forms. Data for the difference in T_1_ in mother-daughter pairs are presented in Figures S4, S5, and S6.

Single mutants (*ace2 ASH1* and *ACE2 ash1*) display a phenotype similar to but less extreme than *ace2 ash1* double mutants (Figure 1D–1G, Table 1). Ace2 contributes to transcriptional activation of *ASH1* [31], so some but not all of the effects of *ACE2* deletion may be a consequence of reduced *ASH1* expression. The characterized indirect effect of Ace2 on DDE sites 5′ of *CLN3* coding sequence [28] likely accounts for at least some of the Ash1-independent effect of *ACE2* deletion; we argue below that there is likely an additional direct effect of Ace2 on *CLN3* transcription.

In strains with *ACE2* and/or *ASH1* deleted, little effect on mother cell size control is expected or observed, since mother cells naturally lack Ace2 and Ash1 due to differential segregation of the factors at cell division (see Introduction). *ace2 ash1* daughters exhibit efficient size control only when born at a size that mothers almost always exceed, due to the budding mode of growth (Figure 1B, 1C) [3].

To test whether Ace2 or Ash1 can affect size control when introduced into mothers, we used mutations resulting in symmetrical inheritance of the factors to mothers and daughters. For Ace2, we used *ACE2-G128E* (indicated as ACE2* from here on). Ace2-G128E accumulates symmetrically and activates Ace2-dependent transcription in both mothers and daughters [20],[32] and was shown previously to reduce mother-daughter G1 asymmetry [28]. For Ash1, we used a mutant (ASH1*) in which mutation of localization elements in *ASH1* mRNA results in accumulation of Ash1 in both mother and daughter nuclei [33].

As with *ace2 ash1* cells, ACE2* ASH1* mothers and daughters that were born at similar sizes exhibited similar αT_1_ values (Figure 2G, 2H; Tables 1, S4). Furthermore, ACE2* ASH1* mothers, when born sufficiently small, exhibit size control, essentially as observed in similarly sized wild-type daughters. Such small mother cells are observed in significant numbers only in glycerol-ethanol culture (Figure 2H).

Thus, making the Ace2/Ash1 daughter-specific gene expression program symmetrical between mothers and daughters (either by deletion or by symmetrical introduction of the factors) results in effective size control (high negative slope in αT_1_ versus ln(M_birth_) plots) over a similar cell size domain in mothers and daughters, eliminating the daughter-specific delay seen in wild-type. In wild-type daughters, size control is exerted at sizes where mothers do not experience size control.

Mother cell size control is in principle hard to detect in any case, because these cells “passed” size control in the previous cycle, and budding yeast cell division removes little or no material from the mother cell. For this reason, even in ACE2* ASH1* cells, which presumably all have daughter-type size control, mothers small enough to allow detection of size control are relatively rare.

Strains in which only Ash1 or Ace2 is symmetrically localized show intermediate phenotypes (Figure 2C–2F; Table 1), suggesting again that both transcription factors contribute to delay in T_1_ in partially independent ways. ACE2* and ASH1* had little effect on size control properties of daughter cells, as expected since these factors are already present in wild-type daughters.

Altogether, these results show that Ace2 and Ash1 define daughter-specific programs that shift size control responses to larger cell size. Ace2 and Ash1 appear to be necessary for this shift in size control in daughters compared to mothers; in addition they are sufficient for imposing daughter-like size control properties when introduced in mothers.

**Figure 3:**
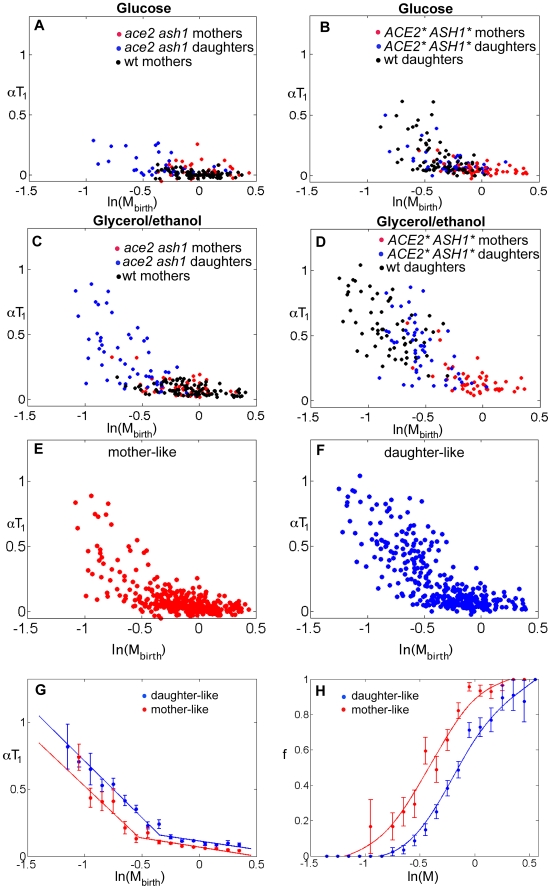
Daughter-specific localization of Ace2 and Ash1 results in asymmetric cell size control. Correlation between αT_1_ and ln(M_birth_) for mothers and “pseudo-mothers” (in (A) cells grown in glucose, in (C) cells grown in glycerol/ethanol) and daughters and “pseudo-daughters” (in (B) cells grown in glucose, in (D) cells grown in glycerol/ethanol). (E) Correlation between αT_1_ and ln(M_birth_) for mothers and “pseudo-mothers” grown in glucose and glycerol/ethanol (pulling together data from (A) and (C)). (B) Correlation between αT_1_ and ln(M_birth_) for daughters and “pseudo-daughters” grown in glucose and glycerol/ethanol (pulling together data from (B) and (D)). (G) Correlation between αT_1_ and ln(M_birth_) in mother-like and daughter-like cells. The graphs are obtained by binning all the data shown in (E) and (F). Error bars are standard errors of the mean. (H) Conditional probability of Whi5 nuclear exit as a function of ln(M) from data in (G). f is the probability that Whi5 will exit the nucleus at size ln(M) given that it had not exited at a smaller size.

These results led to the idea that ACE2* ASH1* mothers should be “pseudo-daughters” with respect to size control, while *ace2 ash1* daughters should be “pseudo-mothers.” To test this, we combined data for mothers and pseudo-mothers, and daughters and pseudo-daughters, in rich and poor medium (Figure 3A–3F). We define mothers and pseudo-mothers as “mother-like,” and daughters and pseudo-daughters as “daughter-like.” Remarkably, these combined data sets collapsed onto one plot for all mother-like cells and a different plot for all daughter-like cells (Figure 3E, 3F). The individual datasets fit well with the average behavior, as shown by plots separating out the various components (Figure S7, S8). The noise about the lines in these plots (size-independent variation) is of a magnitude consistent with previous results (Table S5) [5]. Further analysis showed that the daughter-like plot could be transformed to the mother-like plot simply by shifting the curve 0.2 units of ln(M_birth_) (Figure 3G, 3H). This implies that, with respect to Start, cells containing Ace2 and Ash1 interpret a given cell size as being ∼20% smaller than cells lacking Ace2 and Ash1.

These results can be interpreted in the classical framework of sizers and timers [18],[34] by defining the point at which cells switch from efficient size control to a timer control (the intersection between the two lines fitting the correlation between α*T*_1_ and ln(*M*_birth_) in Figure 3C) as “critical size”: a precise size that cells must attain to transit Start. This analogy is imperfect (the slopes are not −1 or 0, as required for perfect sizers and timers [5],[30], and the sharpness of the transition point cannot be rigorously determined) but provides a useful simplification using the terms of prior size control literature. Using this terminology, the effect of daughter-specific localization of Ace2 and Ash1 is to cause daughter cells to have a larger “critical size” than mother cells (increased by 0.2 units of ln(M_birth_), or ∼20% larger). We emphasize that size control remains highly effective, independent of Ace2 and Ash1; essentially, Ace2/Ash1-containing cells read a given size as smaller than the same size read in the absence of Ace2 and Ash1.

Laabs and collaborators reported symmetrical G1 durations for *ace2* mothers and daughters, and for ACE2* mothers and daughters, independent of cell size [28]. In our experiments, the loss of asymmetrical “interpretation” of cell size caused by these mutations does indeed result in T_1_ durations in mothers and daughters that are more similar than in wild-type (Figure S4, S5). However, our results differ in that in our experiments, size control remains present and effective despite deletion or mislocalization of Ace2 and/or Ash1. As a consequence, the average daughter T_1_ is still significantly longer than the average mother T_1_ (*p* values<10^−3^ in glucose; *p* values<10^−14^ in glycerol/ethanol) even in the mutants, since the budding mode of growth ensures that most daughters are born smaller than most mothers.

This discrepancy likely has a number of sources. First, our use of T_1_, the time from cytokinesis to Whi5 nuclear exit as a landmark, rather than the differential time to budding for mothers versus daughters, as measured by Laabs and collaborators [28], greatly increases the sensitivity with which size control can be detected, since the interval from Whi5 exit to budding is quite variable, cell-size-independent, and very similar in mothers and daughters [5]. Inclusion of this noisy interval blurs the mother-daughter distinction, which is restricted to T_1_. Second, the use of medium supporting slow cell growth (glycerol-ethanol) enhances the ability to detect size control, simply because daughters (of all genotypes) are born much smaller; the work of Laabs et al. [28] employed only rich glucose medium, making size control harder to detect. Our time resolution is also 3 min per frame rather than 10. Finally, our cell size estimates are based on the validated *ACT1-DsRed* marker [5], while Laabs et al. [28] employed volume estimations from geometry of cell images. We have found that the latter method gives on average similar results to *ACT1-DsRed* but increases noise in the detection of size control effects [5].

### Genome-Wide Analysis of Ace2 and Ash1 Targets

*CLN3* was proposed as the relevant indirect transcriptional target of Ace2 to account for mother-daughter asymmetry [28]. Because Ace2 could affect other genes involved in cell size control or mother-daughter asymmetry, and because we had evidence for the involvement of an independent transcription factor, Ash1, we carried out an unbiased search for the transcriptional target(s) through which Ace2 and Ash1 modulate size control in daughters. We performed microarray analysis of synchronized cell populations, comparing cells lacking Ace2 and Ash1 to cells in which they localize symmetrically to both mother and daughter nuclei. Doing the comparisons in this way, rather than simply comparing wild-type to mutants, increases sensitivity of the analysis, since wild-type cultures always contain a mixture of mothers and daughters, reducing the detectable effects of manipulation of daughter-specific transcription factors. Our approach relies on three comparisons: *ace2 ash1* versus ACE2* ASH1*, *ace2* versus ACE2*, and *ash1* versus ASH1* (see Dataset S2 for the microarrays raw data).

We also compared *swi5*, *ace2*, *swi5 ace2*, and wild-type in order to obtain insight into the set of genes regulated by one or both of these factors (see Dataset S1 for the microarrays raw data). Swi5 and Ace2 are closely related transcription factors that recognize the same DNA sequence and share many target genes [35],[36]. The best characterized Ash1 target, *HO*, is also a Swi5 target and its regulation by Swi5 and Ash1 is required for mother-daughter asymmetry in mating type switching [26],[27].

**Figure 4:**
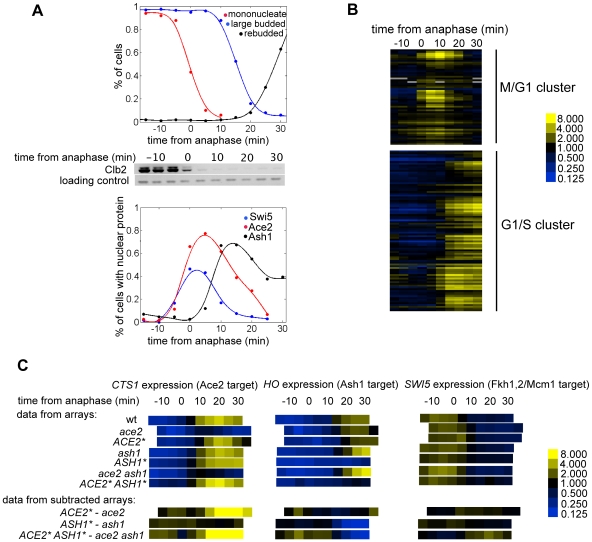
Genome-wide analysis of Ace2 and Ash1 targets. (A) Analysis of cell cycle synchronization and nuclear localization of Ace2, Swi5, and Ash1 in a *cdc20* block-release experiment. Top panel shows the percentage of mononucleate cells, large budded cells, and cells that have rebudded. The middle panel shows the levels of mitotic cyclin Clb2. The lower panel shows the dynamics of nuclear localization of fluorescently tagged Ace2, Swi5, and Ash1. (B) Expression data from the M/G1 and G1/S cell cycle regulated cluster of genes. (C) The regulation of *CTS1* (Ace2 target), *HO* (Ash1 target), and *SWI5* (Fkh1,2 Mcm1 target) expression from the microarray series, as well as data obtained by point-by-point subtraction of the arrays (ACE2*−*ace2*, ASH1*−*ash1*, ACE2* ASH1*−*ace2 ash1*). In these graphs, the time of anaphase, which varies slightly between experiments, was used as the zero time to make the comparisons more accurate.

To synchronize cells during the critical M/G1 interval, we used strains expressing Cdc20 under the control of an inducible promoter (the truncated *GAL1* promoter, *GALL* [37]). Cells were arrested in metaphase by depletion of Cdc20 in glucose medium and released from the arrest by transfer to galactose medium to reinduce Cdc20. This synchronization procedure provides excellent synchrony in M/G1 (anaphase, cell division, and early G1) immediately following release, which is the time of nuclear localization of Ace2, Swi5, and Ash1 (Figure 4A) [36],[38].

About 15 min after release, cells of all genotypes complete anaphase and degrade the mitotic cyclin Clb2 (see Figure 4A). Subsequently, cells separate and rebud (Figure 4A). Both Swi5 and Ace2 enter the nucleus at about the time of anaphase (Figure 4A). On average, Swi5 nuclear entry precedes Ace2 nuclear entry by 2–3 min (see Text S1). A slightly longer (10 min) Ace2 delay relative to Swi5 entry was recently reported [39]. Swi5 is rapidly degraded and disappears before cytokinesis and cell separation (Figure 4A and Text S1) [40]. Ace2 is quickly excluded from the mother nucleus but remains in the daughter nucleus for a significant period during G1 (Figure 4A and Text S1) [20]. Ash1 protein begins to accumulate a few minutes after Swi5 and Ace2 nuclear entry and localizes to the nucleus slightly before cytokinesis, remaining until about the time of budding (Figure 4A and Text S1) [26].

The microarrays for wild-type cells show well-defined M/G1 and G1/S clusters consistent with previous results (Figure 4B) [38]. Furthermore, well-characterized Ace2 and Ash1 targets, such as *CTS1* and *HO*, behave as expected upon transcription factor deletion or mislocalization (see Figure 4C). Cell-cycle-regulated genes that are unaffected by the two transcription factors behave very similarly in all arrays (Figure 4C). Note that the time of anaphase, which varies slightly between experiments, was used as the zero time to make the comparisons more accurate.

The high reproducibility of these microarray data allows us to do a time-point by time-point subtraction of the deletion mutant data from the mislocalization mutant data. This subtraction cancels out cell-cycle-regulated changes in gene expression that are independent of Ace2 and/or Ash1, allowing the hierarchical clustering algorithm [41] to efficiently detect changes that are specifically due to these transcription factors (see Figure 4C).

Clustering analysis of the subtracted data reveals a clear Ace2-dependent cluster composed of well-characterized Ace2-dependent genes, such as *CTS1*, *DSE1*, and *DSE2* (see Text S1 and Figure S1 for a complete list). Only two genes, *HO* and *PST1*, displayed strong changes in expression upon deletion versus mislocalization of Ash1 (see Text S1).

None of the genes whose expression was obviously and strongly Ace2- or Ash1-dependent appeared to be a good candidate to account for daughter-specific regulation of Start. We therefore performed a statistical analysis to obtain a list of genes specifically regulated by both Ace2 and Ash1. We imposed an “AND” logical condition that co-regulated genes should be detected as differential signals in the subtracted *ace2* versus ACE2*, *ash1* versus ASH1*, and *ace2 ash1* versus ACE2* ASH1* comparisons. Additionally, we imposed a temporal requirement that the observed Ace2/Ash1-dependent changes in expression be observed only at times when these factors have accumulated in wild-type nuclei (Figure 4A). This criterion excludes genes whose changes in expression are long-term, indirect consequences of mutation of Ace2 or Ash1. Using a *p* value cutoff sufficient for an expected false positive rate of less than one gene over the whole genome (see Text S1 and Table S3), we identified only five Ace2/Ash1 shared targets: *CLN3*, *HSP150*, *MET6*, *YRF1-1*, and *YRF1-5*.

Direct interactions between Ace2 or Ash1 and the promoters of three of these genes (Ace2 with *CLN3* and *HSP150*; Ash1 with *YRF1-1*) were previously observed in chromatin immuno-precipitation (ChIP)-chip experiments [42],[43], supporting the validity of our analysis (see Text S1).

Prominent in the list of genes affected by both Ace2 and Ash1 is the G1 cyclin, *CLN3*, a rate-limiting activator of the Start transition. Laabs and collaborators had likewise implicated *CLN3* as a gene repressed by Ace2, based on comparing *CLN3* RNA levels with and without Ace2, and examining mother versus daughter accumulation of GFP driven from a truncated *CLN3* promoter [28]. In that paper, it was also suggested that Ace2 might regulate *CLN3* indirectly through an unknown transcription factor that represses *CLN3* expression in daughters by binding to DDE sites on the *CLN3* promoter. Among all the identified Ace2 targets, Ash1 is the most likely candidate transcription factor for a repressive role on *CLN3* expression. We observe, however, that there is no obvious homology between the Ash1 consensus and the DDE. In the next sections we provide evidence that Ash1 binds to the *CLN3* promoter and that this binding is at least in part mediated by Ash1 consensus-binding sites that are different from the DDE.

Together, these findings suggested the hypothesis that differential regulation of Start in mothers and daughters due to Ace2 and Ash1 may be solely a consequence of differential regulation of *CLN3*.

**Figure 5:**
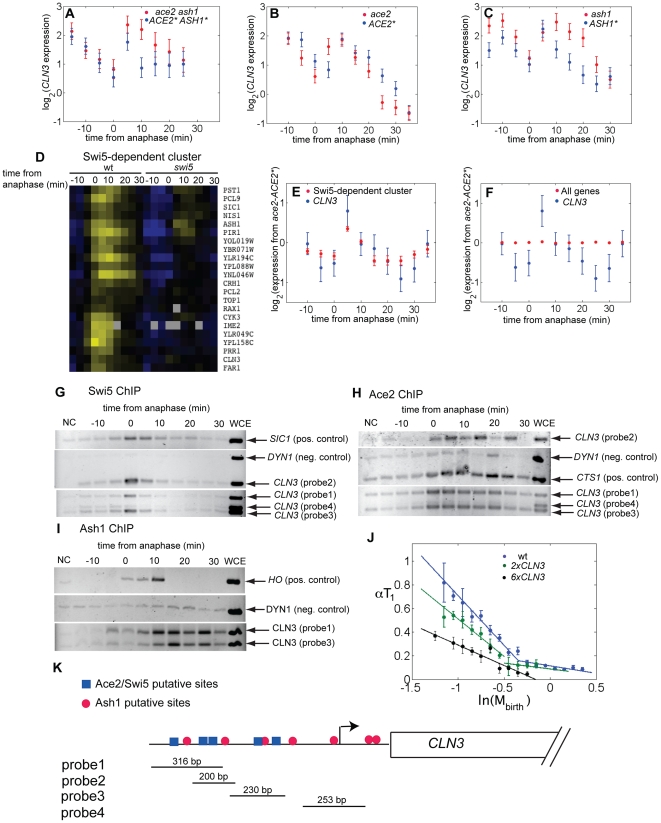
Ace2, Swi5, and Ash1 regulate the expression of the G1 cyclin *CLN3*. *CLN3* expression: (A) ACE2* ASH1* versus *ace2 ash1*, (B) ACE2* versus *ace2*, (C) ASH1* versus *ash1*. The error bars were estimated from the variability in expression of the large number of genes that are not affected by Ace2 and Ash1. The expression levels of *CLN3*, as well as any other gene in the genome, were estimated by at least four measurements from four distinct probes. These measurements showed a smaller variability than the presented error bars, suggesting that the reported error bars are a conservative estimate of measurement errors. (D) Expression of a cluster of Swi5-dependent genes. Arrays from *GALL-CDC20* block release time course experiments of wild-type and *swi5* cells were hierarchically clustered. A Swi5-specific cluster is shown (see Text S1 for a complete list of genes regulated by Swi5). (E) *CLN3* expression compared with the average expression of the remaining genes belonging to the Swi5-specific cluster (error bars indicate s.e.m.) from the dataset obtained by subtracting the ACE2* data from the *ace2* data. (F) *CLN3* expression compared with the average expression of the whole genome (error bars indicate s.e.m.) from the same dataset (i.e. *ace2*−ACE2*). ChIP analysis of the interaction between Swi5 (G), Ace2 (H), Ash1 (I), and the *CLN3* promoter. Following cross-linking and immunoprecipitation, DNA was amplified by PCR. Amplification of a region of the ORF of *DYN1* was used as negative controls, while regions of the *SIC1*, *CTS1*, and *HO* promoters were used as positive controls for Swi5, Ace2, and Ash1, respectively. All the strains were TAP-tagged (NC, negative control from an untagged strain; WCE, whole cell extract). The ChIP data were reproduced for Ace2 and Ash1. The Swi5 data are from a single experiment. (J) Correlation between αT_1_ and ln(M_birth_) in daughter cells carrying different copy numbers of *CLN3*. (K) Representation of the Ace2/Swi5 and Ash1 putative binding sites on the *CLN3* promoter.

*CLN3* expression in M/G1 is from 1.5- to 2.5-fold higher in *ace2 ash1* cells (pseudo-mothers) than in ACE2* ASH1* cells (pseudo-daughters) (Figure 5A), suggesting that *CLN3* is differentially regulated in wild-type mothers and daughters. Previously published data support this idea: in populations of cells containing both mothers and daughters, *CLN3* expression peaks at the M/G1 boundary [44], while in populations of size-selected daughters *CLN3* expression peaks later in G1 [45], or shows no peak [12],[46], consistent with our conclusion that *CLN3* expression in M/G1 is higher in mothers than in daughters. M/G1 expression of *CLN3* is driven by Mcm1 through early cell-cycle box (ECB) elements [44]; our results and the results of Laabs and collaborators [28] suggest that in daughters, Ace2 and Ash1 antagonize this activation.

Hierarchical clustering of microarrays of wild-type and *swi5* cells indicates that *CLN3* belongs to a cluster of genes whose expression is activated by Swi5 (Figure 5D). Analysis of *ace2* versus ACE2* arrays (Figure 5B) shows that *CLN3* behaves similarly to the rest of this Swi5 dependent cluster upon manipulation of *ACE2* (see Text S1 and Figure S1 for a complete list of Swi5 and Ace2 targets). Expression of these genes in ACE2* cells is lower than expression in *ace2* at 5 min after anaphase, but higher from 15 min to 25 min (Figure 5E); that is, the genes appear to be repressed by Ace2 at early times, then activated by Ace2 at later times. This pattern is significantly different from a pattern assuming no regulation by Ace2 (*p*<10^−11^). *CLN3* expression depends on Ace2 similarly to these other Swi5 targets (probability that *CLN3* is regulated as the other Swi5/Ace2 targets: *p* = 0.7, Figure 5E; a model assuming that *CLN3* is not affected by Ace2 can be excluded with *p*<0.03, Figure 5F).

Thus *CLN3* and a class of Swi5 dependent genes follow a pattern consistent with early repression and late activation by Ace2, and with early activation by Swi5, likely acting in concert with ECB regulation [44]. We do not know the mechanism underlying this complex pattern. We speculate that Ace2 may be an intrinsically poorer activator than Swi5, but it activates for a longer period due to its longer nuclear residence. Swi5 disappears from both mother and daughter nuclei a few minutes after anaphase, while Ace2 persists in daughter nuclei for about 20 min longer (Figure 4A). Competition between Ace2 and Swi5 for the same binding site [35] could then contribute to the differential expression observed in these arrays. Alternatively, Ace2 could directly repress expression of these genes; however, no previous evidence suggests a directly repressive role for Ace2.

Microarray analysis for *ash1* and ASH1* shows that *CLN3* expression is repressed about 2-fold by Ash1 during the period from 10 min to 25 min after anaphase (Figure 5C). During this interval Ash1 is present in the nucleus (Figure 4A), suggesting that it could be a direct repressor of *CLN3* expression.

Many Swi5 and Ace2/Swi5 targets have moderately higher expression in the absence of Ash1 (Figure S2). The absolute repression of Swi5-dependent *HO* expression by Ash1 in daughter cells may thus be an enhancement of a common pattern of co-regulation.

Our data suggest that Ace2 and Ash1 cooperate to repress *CLN3* expression in daughters. Consistently, activation of the G1/S regulon controlled by Cln3 is delayed and/or happens at larger cell size in *cdc20*-synchronized cells containing these factors (Figure S3).

### Ace2, Swi5, and Ash1 May Be Direct Transcriptional Regulators of *CLN3*

We performed chromatin immuno-precipitation (ChIP) experiments in synchronized cell populations to ask if Ace2, Swi5, and Ash1 bind to the *CLN3* promoter. Genome-wide localization data in asynchronous cell populations suggested binding of Ace2 and Swi5 to the *CLN3* promoter but were statistically insufficient to definitively prove the association [42],. We used synchronized cell populations to provide dynamic information on the possible binding of Ace2, Swi5, and Ash1 to the *CLN3* promoter, providing a higher signal to noise ratio than can be obtained from asynchronous cells.

Swi5 and Ace2 bound to regions in the *CLN3* promoter around the time of anaphase, coincident with their nuclear entry (Figure 5G, 5H). Swi5 is on the *CLN3* promoter for only a few minutes (Figure 5G), while Ace2 is on the *CLN3* promoter for about 20 min (Figure 5H), also consistent with the time of Swi5 and Ace2 nuclear localization (Figure 4A and Text S1). Thus, Ace2 and Swi5 might directly regulate *CLN3* transcription by binding to multiple Ace2/Swi5 sites in the *CLN3* promoter.

A previous meta-analysis of multiple ChIP-chip experiments concluded that Swi5 and Ace2 both bound the *CLN3* promoter with high probability (data in Supp. Table S5 of Ref. [47]), consistent with our results.

Ash1 binds the *CLN3* promoter with kinetics similar to its nuclear localization (Figure 5I and Figure 4A). In contrast, Ash1 residence at the *HO* promoter is much briefer, consistent with previous results [20], despite persistence of Ash1 in the nucleus. We do not know the reason for this difference.

### Mutations of Ace2/Swi5 and Ash1 Binding Sites on the *CLN3* Promoter Reduce the Asymmetry of Start Regulation

**Figure 6:**
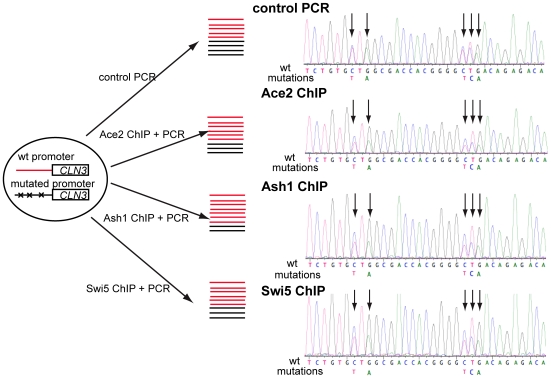
Binding of Ace2, Swi5, and Ash1 to the *CLN3* promoter is reduced by mutation of the Ace2/Swi5 and Ash1 consensus-binding sites. Experimental strategy to estimate the preferential binding of Ace2, Swi5, and Ash1 to their consensus-binding sites. Following ChIP, various regions of the *CLN3* promoter were amplified by PCR and analyzed by sequencing to obtain an estimate of the ratio of wild-type promoter sequences to mutated sequences. This ratio compared to the same ratio from PCR of genomic DNA provides an indication of the preferential binding of the factors to these sequences (Table 2).

We noted three candidate Ace2/Swi5 sites (GCTGGS, consensus sequence: GCTGGT; [42]) in the *CLN3* promoter. The *CLN3* promoter also contains two possible variant sites (GCTGA); such sites are over-represented in Ace2 and Swi5 targets (B.F., unpublished data). There are eight candidate Ash1-binding sites (YTGAT) [48] in the *CLN3* promoter. We mutated these Ace2/Swi5 and/or Ash1 putative binding sites in the *CLN3* promoter by exact gene replacement (see Text S1 for details). To test if Ace2, Swi5, and Ash1 bind to these sites, we performed ChIP analysis in synchronized populations of heterozygous diploid strains containing a wild-type copy and a mutated copy of the *CLN3* promoter (Ace2/Swi5 and Ash1 putative binding sites mutated). Following immunoprecipitation, various regions of the *CLN3* promoter were amplified by PCR and analyzed by sequencing to obtain an estimate of the ratio of wild-type promoter sequences to mutated sequences (Figure 6). These experiments are internally controlled (as they do not require the comparison of two independent ChIP experiments). The measured ratio provides an indication of the preferential binding of Ace2, Swi5, and Ash1 to the identified putative binding sites. Ace2, Swi5, and Ash1 binding to the mutated *CLN3* promoter was reduced to about 60% relative to the wild-type promoter, assaying multiple sequences from −1,183 to −998 (ATG: +1) (Table 2 and Table S6). The binding of Ace2, Swi5, and Ash1 to sequences from −767 to −545 is not altered by mutation of the putative binding sites (Table 2 and Table S6). These results indicate that we have identified authentic Ace2, Swi5, and Ash1 binding sites in the 5′ region of the *CLN3* promoter. The residual binding signal from the mutant is consistent with either a low level of background precipitation, or to genuine residual binding of the factors to non-consensus sites in the promoter. (Due to uncertainties about such other sites, as well as variable shearing of the DNA in the ChIP procedure, we do not think we can use these data to reliably map which candidate site(s) might be directly bound by Ace2, Swi5, or Ash1).

**Table 2:** Ace2, Ash1, and Swi5 Binding to the *CLN3* Promoter in Heterozygous Diploids.

	Ace2	Ash1	Swi5
Binding ratio (−1,183: −998)	0.66±0.03 (<10^−20^)	0.60±0.05 (<10^−20^)	0.74±0.05 (<10^−5^)
Binding ratio (−767: −545)	1.15±0.16 (0.34)	1.01±0.16 (0.94)	1.15±0.14 (0.29)

The ratio of binding of Ace2, Ash1, and Swi5 to mutated and wild-type *CLN3* sequences in heterozygous diploids is reported (in parenthesis is the *p* value that the measured ratio is compatible with no change in binding). Binding to the *CLN3* promoter region between −1183 and −998 (ATG: +1) is significantly reduced upon mutation of the putative Ace2/Swi5 and Ash1 binding sites (see Table S6 for details). Binding to the region from −767 to −545 is not affected.

As a test to see if we might have missed significant binding sites in the *CLN3* promoter, we carried out a bioinformatics analysis (Text S1 and Figure S11) looking for regulatory motifs in the promoters of the identified Ace2 and Swi5 targets in S. cerevisiae and three closely related yeasts. Interestingly, we found only two strongly conserved sites: one was one of the candidate Ace2/Swi5 sites we mutated, at position −701, and the other was a similar but non-consensus site (GCTTGG) at position −569, which we did not mutate since it did not meet the consensus we used in designing the mutagenesis (see above). It is possible, although still untested, that this non-consensus site could account for residual binding of Ace2 to the mutant promoter.

The absence of a cluster of Ash1-dependent genes and the low information content of the known Ash1 consensus site (YTGAT) does not allow us to perform similar bionformatics analysis; therefore, we cannot test the hypothesis that there are non-consensus Ash1 sites in the *CLN3* promoter that we did not mutagenize.

**Figure 7:**
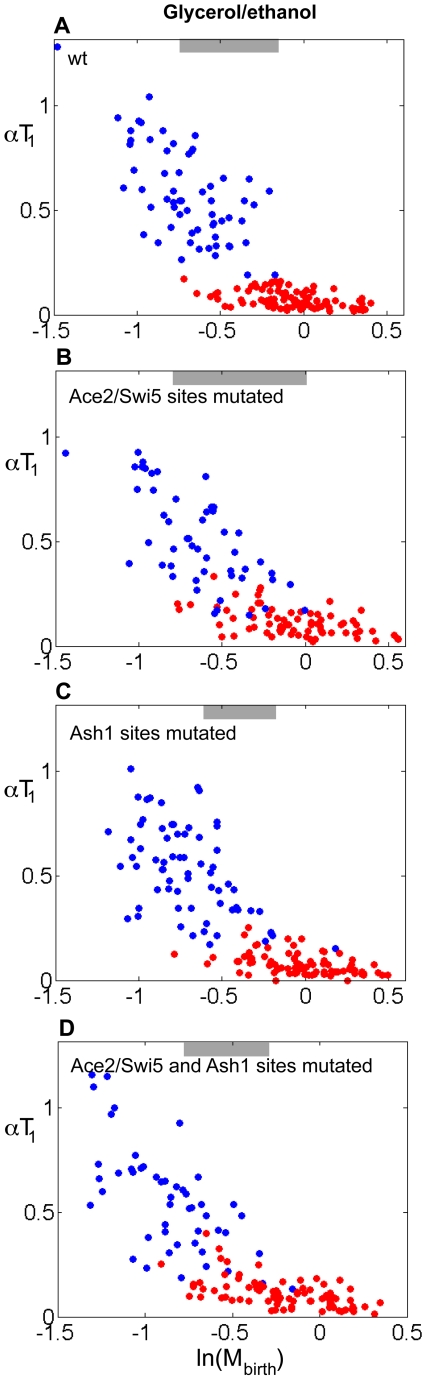
Mutation of the Ace2/Swi5 and Ash1 binding sites on the *CLN3* promoter reduces the asymmetrical regulation of Start. Correlation between αT_1_ and ln(M_birth_) for cells grown in glycerol/ethanol in mutants lacking the Ace2/Swi5 and/or Ash1 sites on the *CLN3* promoter. (A) wild-type, (B) Ace2/Swi5 sites mutated, (C) Ash1 sites mutated, (D) Ace2/Swi5 and Ash1 sites mutated. Red dots, mothers; blue dots, daughters. Gray bars indicate the region of size overlap used for the analysis presented in Table 1.

To test if the reduced binding of Ace2, Swi5, and Ash1 to the *CLN3* promoter also has an effect on the regulation of Start, we analyzed the correlation between αT_1_ and ln(M_birth_) in strains carrying mutations of the identified Ace2/Swi5 and/or Ash1 putative sites. These plots show that these mutations reduce the T_1_ delay in daughters compared to similarly-sized mothers (Figure 7, Table 1). The effect is easily detected and statistically significant in cells grown in glycerol-ethanol (Figure 7), a similar effect was observed in glucose, but this effect did not reach nominal statistical significance (Figure S9). In the Ace2/Swi5 sites mutant (Figure 7B, 7D) the duration of T_1_ in mothers is prolonged, consistent with the idea that Swi5 is an activator of *CLN3* (since mothers do not contain Ace2). Simultaneous mutation of Ace2 and Ash1 sites did not significantly enhance the phenotype of mutation only of one or the other (Figure 7, Table 1).

Although these promoter mutations have significant effects, they are less potent than deletion of *ACE2* and *ASH1* (compare Figure 1 with Figure 7, see Table 1). This may be in part due to the presence of additional non-consensus Ace2/Swi5 or Ash1 sites in the *CLN3* promoter (discussed above). Additionally, the comparison between mutating Ace2 sites and deleting *ACE2* is not exact because removing Ace2 sites perforce also removes Swi5 sites, and on the other hand, deletion of Ace2 alters *ASH1* expression.

The promoter mutants could also be less effective than deletion of *ACE2* and *ASH1* because Ace2 has indirect effects on *CLN3* expression. It was previously shown that “DDE” sites in the *CLN3* promoter play an important role in Ace2-dependent asymmetric control of Start, but these sites did not appear to be bound by Ace2, suggesting an indirect mechanism [28]. Interestingly, these sites are transcribed into the *CLN3* mRNA, and the Whi3 RNA binding protein binds to a repeated RNA sequence at the center of the DDE [49]. Whi3 is a regulator of cell size control thought to work by regulation of *CLN3* mRNA and protein [49],[50],[51],[52],[53]. At present, though, there is no information implicating Whi3 in mother-daughter asymmetry, nor is Ace2 known to regulate Whi3.

Finally, Ace2/Ash1 could regulate additional G1-regulatory genes at a level not detectable by our statistical analysis (see above).

The observation that reduced binding of Ace2, Swi5, and Ash1 to the *CLN3* promoter results in a significant reduction of asymmetric control of Start by cell size in mothers and daughters supports the idea that Ace2 and Ash1 directly repress *CLN3* expression in M/G1, accounting for a significant part of the regulation of G1 length by these transcription factors. We suggest that direct regulation of *CLN3* by Ace2 and Ash1 together with its indirect regulation by Ace2 through the DDE sites [28] can explain asymmetric control of Start by cell size in mothers and daughters.

### Changes in *CLN3* Expression Are Sufficient to Account for Mother-Daughter Asymmetry

*CLN3* expression in M/G1 is ∼2-fold higher in *ash1 ace2* cells (pseudo-mothers) than in ASH1* ACE2* cells (pseudo-daughters) (Figure 5A). While this change is small, *CLN3* is a highly dosage-sensitive activator of Start. Previous measurements of cell sizes in cycling cell populations demonstrated effects on cell size upon 2-fold changes up or down in *CLN3* gene dosage [10],[44].

To increase the precision of this analysis, we analyzed the correlation between αT_1_ and ln(M_birth_) in cells carrying either two or six copies of *CLN3*. This focuses the analysis on the critical interval, since Cln3 decreases cell size specifically by decreasing T_1_ in wild-type mothers and daughters [5].

Daughter cells with two copies of *CLN3* exhibit efficient size control [high negative slope in αT_1_ versus ln(M_birth_)] over a size range that is shifted to smaller cell size by ∼0.15 units of ln(M_birth_), compared to wild-type; this shift is similar to that in *ace2 ash1* daughter cells (mother-like cells) (compare Figure 5J to Figure 3C). Thus, the observed ∼2-fold changes in *CLN3* expression upon deletion versus mislocalization of Ace2 and Ash1 could account for the observed changes in cell size control in these mutants.

Six copies of *CLN3* almost eliminate size control even in very small daughter cells (Figure 5J). Thus, size control is remarkably sensitive to *CLN3* gene dosage; it can only be modulated by altering *CLN3* expression in a narrow range before size control is lost.

### Asymmetric Regulation of *CLN3* Is Required for Asymmetric Regulation of Start

We analyzed the correlation between αT_1_ and ln(M_birth_) in *cln3* cells and in *cln3* cells expressing the *CLN3* ORF (without the upstream DDE sites) from constitutive promoters. It is important for this analysis that the constitutive promoters provide expression levels of Cln3 similar to those in wild-type cells and that the promoter-*CLN3* fusions complement the large-cell phenotype of *cln3* mutants, without “overshoot” to a small-cell phenotype [8],[10],[54]. We screened a number of different constitutive promoters of different strengths [55] for these properties, examining both cell size and Cln3 protein levels using myc-tagged Cln3, compared to wild-type (including an approximate correction for cell cycle regulation of *CLN3* expression from the endogenous promoter) (Table 3; [44]).

**Table 3:** Levels of Cln3 expression and average cell size for asynchronous cell populations expressing *CLN3* from various constitutive promoters.

	Wild-Type	*cln3*	*CDC28pr-CLN3*	*ACT1pr-CLN3*	*ADH1pr-CLN3*
Cln3 levels in D	1	0	0.4–0.6	5–7	8–10
Cln3 levels in g/e	1	0	0.2–0.5	8–10	1.5–2.0
Cell size in D (fl)	56	92	84	45	45
Cell size in g/e (fl)	47	88	60	41	51

The expression of *CLN3* is cell cycle regulated with a peak in expression at M/G1 characterized by a peak to trough ratio of order 3 (see Figure 5) [44]. Since the period of peak expression is brief, we consider a construct yielding ∼3 times the average expression level of endogenous Cln3 to give approximately wild-type levels of expression during the critical interval.

The *ACT1* and the *ADH1* promoters result in over-expression of Cln3 and in a small cell-size phenotype for cells grown in glucose-containing media (Table 3). Expression of Cln3 from the *CDC28* promoter is weaker than expression from the *CLN3* promoter and results in cell sizes bigger than wild-type and only slightly smaller than *cln3* cells (Table 3). Integration into the yeast genome of six copies of the *CDC28pr-CLN3* construct results in a cell size distribution similar to that of wild-type cells. We also analyzed the effects of these constructs in glycerol-ethanol medium. Four tandemly integrated copies of *CDC28pr-CLN3* results in an overall cell size distribution similar to that of wild-type cells in glycerol-ethanol (Table 3). As a result of decreased *ADH1* expression in non-fermentable media [56], the *ADH1* promoter provides Cln3 levels similar to endogenous levels in glycerol-ethanol, resulting in a cell size distribution slightly (∼10%) larger than wild-type (Table 3).

**Figure 8:**
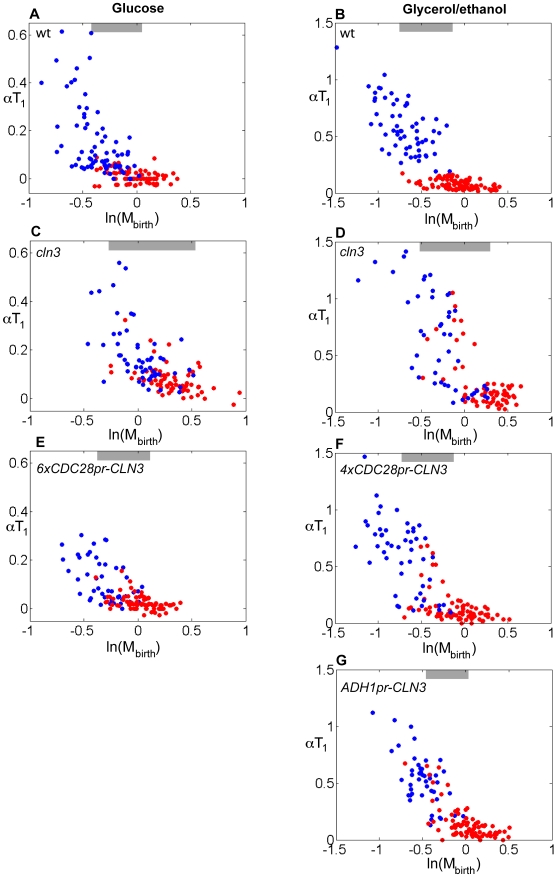
Symmetric regulation of *CLN3* expression result in symmetric control of Start in mothers and daughters. Correlation between αT_1_ and ln(M_birth_) for cells grown in glucose or glycerol/ethanol. (A–B) wild-type, (C–D) *cln3*, (E) *cln3 6xCDC28pr-CLN3*, (F) *cln3 4xCDC28pr-CLN3*, (G) *cln3 ADH1pr-CLN3*. Red dots, mothers; blue dots, daughters. Gray bars indicate the region of size overlap used for the analysis presented in Table 1.

Measurements of Cln3 protein levels show that Cln3 overexpressors were smaller than wild-type, and underexpressors larger (Table 3); therefore, the measurements of Cln3 level were accurate over a physiologically relevant range. Based on results with a single copy of *CDC28pr-CLN3-myc*, four to six copies of *CDC28pr-CLN3* should produce approximately wild-type levels of Cln3 in M/G1, consistent with the observed cell size distributions (Table 3 and Figure 8).

We therefore used strains containing 6*xCDC28pr-CLN3* in glucose medium, and strains containing 4*xCDC28pr-CLN3* or *ADH1pr-CLN3* in glycerol-ethanol medium, to provide approximately endogenous levels of expression without mother-daughter asymmetry (and presumably without regulation by the cell cycle, Ace2, or Ash1; note that the 5′ DDE sites are not present in these constructs). In 6*xCDC28pr-CLN3* cells the daughter-specific delay is almost entirely abolished (Figure 8C, 8E and Table 1). Similarly, in 4*xCDC28pr-CLN3* and *ADH1pr-CLN3* cells grown in glycerol/ethanol, the daughter-specific delay is almost entirely abolished, and small mothers and daughters have similar size control properties (Figure 8D, 8F, and 8G and Table 1). Thus, similarly to the results obtained by placing Ace2 and Ash1 in mother nuclei, size control in small mother cells can be detected by eliminating differential mother-daughter control of *CLN3* expression.

Small *4xCDC28pr-CLN3* and *ADH1pr-CLN3* cells in glycerol/ethanol exhibit strong size control (slopes of ∼−0.8, compared to a theoretical expectation for perfect size control of −1 [5],[30]) (Figure 8F, 8G), suggesting that while daughter-specific transcriptional regulation of *CLN3* specifies the cell size domain over which size control is effective, the intrinsic mechanism of size control is not dependent on mother-daughter regulation of *CLN3* transcription, or indeed on any transcriptional regulation acting through the *CLN3* promoter. We speculate that an M/G1 burst of *CLN3* expression from Mcm1 and/or Swi5 ([44]; Figure 5) may be sufficient to drive cells rapidly through T_1_, as is observed in wild-type mothers of all sizes (Figure 1B, 1C; [5]); in daughters, this burst may be suppressed by Ace2 and Ash1.

The daughter-specific delay of wild-type cells depends on *CLN3*, since *cln3* mother and daughter cells of similar size have similar αT_1_. Remarkably, cells deleted for *cln3* still exhibit strong effects of cell size on G1 duration, although these effects are symmetrical between mothers and daughters of similar size (Figure 8C, 8D). Thus, while the cell size domain of effective size control in wild-type cells is set by *CLN3*, there may be an underlying Cln3-independent or parallel program of cell size control that acts or becomes detectable only upon deletion of *CLN3* [57]. In addition to loss of mother-daughter asymmetry, the response of *cln3* cells to cell size is shifted to about 1.5-fold larger cell sizes as measured using *ACT1pr-DsRed*; this finding confirms that *cln3* cells are larger in terms of protein content than wild-type, in contrast to the proposal that the increase in cell size of *cln3* cells [8],[10] is due primarily or entirely to increased vacuole size [58].

Our results are consistent with those of Laabs and collaborators, who reported that *cln3* cells and cells expressing *CLN3* from ectopic promoters lost mother-daughter asymmetry [28]. They also observed equal G1 durations for individual mother/daughter pairs [28]. In our analysis, in contrast, in almost all *cln3* mother-daughter pairs, with or without ectopic expression of *CLN3*, the daughters had a longer T_1_ period (see Figure S6; *p* values<10^−5^ in glucose; *p* values<10^−15^ in glycerol/ethanol), although the daughter delay was reduced compared to wild-type, consistent with the results of Laabs and collaborators [28]. The symmetry that we observe in these mutants is restricted to mothers and daughters of similar size (more precisely, in the mother and daughter plots of αT_1_ versus ln(M_birth_), in regions where the domains of mothers and daughters overlap). We assume this discrepancy arises from the same reasons discussed above.

## Discussion

### Cln3, Size Control, and Asymmetric Transcription

It was previously reported that asymmetric localization of Ace2 represses *CLN3* in daughter cells [28]. Our results extend this finding by showing that Ace2 regulation of *CLN3* is in part direct, mediated by Ace2 binding to the *CLN3* promoter. In addition, our results implicate Swi5 and Ash1 as well as Ace2 in *CLN3* regulation.

Neither asymmetric expression of *CLN3*, nor *CLN3* itself, is essential for size control [57] (Figure 8). However, *CLN3* sets the domain of cell sizes over which effective size control operates in wild-type cells. For this reason, negative control of *CLN3* by Ace2 and Ash1 allows differential Start regulation in mothers and daughters.

These findings provide empirical validation for one part of the theoretical cycle of transcriptional regulators proposed to account for a B-type cyclin-independent autonomous transcriptional oscillator [47].

### Do Mothers Drive Start with a Burst of *CLN3*?

In the budding mode of growth, cell mass produced after budding goes to the daughter, but all pre-budding mass is retained by the mother [3]. As a result, a daughter that “passes” size control will retain this size through all subsequent (mother) budding cycles. This cell could thus be accelerated through Start by the M/G1 *CLN3* burst without a “need” for size checking. Given the high amount of noise in the mechanism of size control [5], this could prevent unnecessary delays in already full-sized mothers. The M/G1 *CLN3* burst, if experienced by daughters, would perturb the ability of daughters to effectively check their size (Figure 5J). This could result in the requirement for daughter-specific blockage to the burst. Thus, mother/daughter-specific *CLN3* regulation could simultaneously prevent unnecessary mother delays and prevent smaller daughters from passing Start prematurely.

In addition to repressing initial expression of *CLN3* in M/G1, Ace2 also induces *ASH1* expression; Ash1 represses later expression of *CLN3*. This is an example of “feed-forward” regulation, which may be a common regulatory structure for providing delayed response [59]—in this case, prolonged *CLN3* repression even after loss of Ace2 from the daughter nucleus. We speculate that this mechanism may allow daughter-specific delay over a broad range of timescales and growth rates.

The *CLN3* upstream region is unusually large (1.2 kb, compared to an average intergenic distance of 0.6 kb) and contains six ECB sites [45], multiple Ace2/Swi5 and Ash1 sites (this work), and four DDE sites [28]. How all of these sites and the factors that bind to them cooperate combinatorially to properly regulate *CLN3* is unknown. This is a large amount of regulatory machinery to provide a maximum peak-to-trough ratio only on the order of three [44]; however, since manipulation of *CLN3* gene copy number up or down by only a factor of two yields significant cell size phenotypes (Figure 5J) [10],[44], this level of control is likely to be physiologically significant, perhaps for the reasons cited above.

### Sizers, Timers, and Start

As noted above, our results can be interpreted in the classical framework of sizers and timers [18],[34] by defining the point at which cells switch from efficient size control to a timer control as “critical size” (Figure 3C): a precise size that cells must attain to transit Start. This point is marked by the intersection of the line of high negative slope with the line of low or zero negative slope in αT_1_ versus ln(M_birth_) plots. Using this framework, we can summarize our results by stating that Ace2/Ash1-containing cells have a larger “critical size” than cells lacking these factors (normally, daughters and mothers, respectively). This formulation is inexact, primarily due to the evident non-zero slope in our data for the second component of the two-slope fit; remarkably, though, the effects of Ace2 and Ash1 shift not just the intersection point but the entire curve by 0.2 units of ln(M_birth_).

While increasing cell size and increasing Cln3 both decrease T_1_ (i.e., accelerate Whi5 exit from the nucleus after cytokinesis) [5], Ace2 exit from the daughter nucleus occurs about 15 min (15±6 min) after cytokinesis, independent of cell size and *CLN3* (Text S1 and Figure S10). Thus, overall, Start control may consist of three distinct modules: Ace2 and Ash1-dependent but cell-size independent setting of the domain of cell size control; size control itself, leading to initiation of Whi5 nuclear exit; and a final size-independent step driven by *CLN1,2-*dependent transcriptional positive feedback, which rapidly completes Whi5 exit and drives the downstream events of Start [5],[7].

### A New Link between Differentiation and Cell Cycle in Budding Yeast

In wild-type homothallic budding yeast, only mother cells express the *HO* endonuclease and switch mating type, due to Ash1 repression of *HO* expression in daughters [26],[27]. Phylogenetic analysis shows that in fungi, *ASH1* appeared before *HO*. This suggests that Ash1 may have functions predating *HO*, which may be important for asymmetrical cell division. It would be interesting to test whether Ash1 functions in cell cycle control in other fungi that can divide asymmetrically, such as *Candida albicans*, which lacks a *HO* homolog but expresses an Ash1 homolog that localizes specifically to the daughter cells [60],[61]. Ash1 also is found in *A. gossypii*, which undergoes asynchronous division in a multinucleate syncitium [62]; it would be interesting to evaluate the role of Ash1 in this asynchrony. Ace2 controls genes that confer diverse aspects of daughter cell biology [20],[23],[24]; here we elucidate how Ace2 also contributes to differential Start regulation in daughters [28].

There are interesting parallels and connections between *HO* control and *CLN3* control. Both are activated by Swi5 and inhibited by Ash1. Swi5 regulation of *HO* in mothers can be interpreted as feed-forward control, since Swi5 directly primes *HO* for expression [63] and also activates *CLN3* expression, which later yields efficient activation of the SBF factor that drives *HO* transcription [7],[63].

Cell cycle regulation and cell differentiation, often driven by asymmetric localization of cell fate determinants during cell division [64],[65],[66], are inter-regulated in many systems [67],[68],[69]. As the decision of cells to differentiate is often made in G1, cell differentiation and commitment to a stable G1 are often coregulated [67],[69],[70]. It would be interesting to examine cases in which stem cells produce one proliferating cell and one daughter that differentiates in G1 [65]; such cells might employ mechanisms similar to those we have uncovered in differential mother-daughter G1 control in budding yeast.

## Materials and Methods

### Strain and Plasmid Construction

Standard methods were used throughout. All strains are W303-congenic. All integrated constructs were characterized by qPCR. Mutations of the Ace2/Swi5 and Ash1 binding sites on the *CLN3* promoter were verified by sequencing.

### Time-Lapse Microscopy

Preparation of cells for microscopy and time-lapse microscopy were performed as previously described [5],[6]. Growth of microcolonies was observed with fluorescence time-lapse microscopy at 30°C using a Leica DMIRE2 inverted microscope with a Ludl motorized XY stage. Images were acquired every 3 min for cells grown in glucose and every 6 min for cells grown in glycerol/ethanol with a Hamamatsu Orca-ER camera. Custom Visual Basic software integrated with ImagePro Plus was used to automate image acquisition and microscope control.

### Image Analysis

Automated image segmentation and fluorescence quantification of yeast grown under time-lapse conditions and semi-automated assignment of microcolony pedigrees were performed as previously described [6]. The nuclear residence of Whi5-GFP was scored by visual inspection of composite phase contrast-fluorescent movies. Cell size was measured as the total cell fluorescence from DsRed protein, expressed from the constitutively active *ACT1pr*, as previously described [5]. Cell size at every time point was extrapolated from a linear fit of the ln(M) as a function of time for cells grown in glucose and from a smoothing spline fit for cells grown in glycerol/ethanol. Individual cell growth in glycerol/ethanol appears to be intermediate between a linear and an exponential model (unpublished data); this deviation from exponentiality has very little effect on this analysis.

### Data Analysis

Time-lapse fluorescence microscopy, microarray data, and sequencing data were analyzed with custom software written in MATLAB software (see Text S1 for details on the analysis of the microarray data) [5]. For cluster analysis, the log_2_ of the arrays data or of the subtracted arrays data were hierarchically clustered by the agglomerative algorithm [41]. Data were visually presented using JavaTreeView. For sequencing data, the area associated to every wild-type or mutated nucleotide was evaluated manually by using the MATLAB software.

### Cell Cycle Synchronization

YEP medium was used for all cell cycle synchronization experiments, supplemented with the appropriate carbon source as indicated below. Cell cycle synchronization by the *cdc20 GALL-CDC20* block release was achieved by growing cells to early log phase in YEP+galactose (3%), then filtering and growing them in YEP+glucose (2%) for 3 h to arrest cells in metaphase. Cells were released from the block by filtering back into YEP+galactose (3%). *GALL* is a truncated version of the *GAL1* promoter that shows inducible but significantly lower expression than the full-length *GAL1* promoter [37].

### Microarrays

Microarrays were performed as previously described [71] but using microarrays carrying PCR fragments from open reading frames of *S. cerevisiae*. Each array had each PCR fragment independently spotted four to eight times, leading to a high redundancy of data and small errors in expression ratios. RNA extraction, cDNA synthesis and labeling, and hybridization and scanning were carried out by the Stony Brook spotted microarray facility, as described previously [71].

### ChIPs

Standard methods were used for ChIP experiments. Early log phase cells were fixed for 15 min in 1% formaldehyde at room temperature. Immunoprecipitations were performed with IgG Sepharose beads. Immunoprecipitated DNA was amplified by PCR.

## Supporting Information

Dataset S1 Microarrays data of wild-type, *ace2*, *swi5*, and *ace2 swi5* synchronized cell populations.
(1.48 MB TXT)

Dataset S2 Microarrays data of wild-type, *ace2 ash1*, ACE2* ASH1*, *ace2*, ACE2*, *ash1*, ASH1* synchronized cell populations.
(2.42 MB TXT)

Figure S1 Hierarchical clustering analysis of genes regulated by Ace2 and Swi5.
(9.90 MB TIF)

Figure S2 Ash1 is a modulator of Swi5-dependent expression. Average expression for Ace2/Swi5 and Swi5 targets (45 genes) in response to Ash1 (data were obtained by subtracting the ASH1* dataset from *ash1* dataset). This graph shows that Ash1 weakly represses the expression of many Ace2/Swi5 and Swi5 targets in daughter cells.
(0.87 MB TIF)

Figure S3 Activation of SBF and MBF is delayed by Ace2 and Ash1. Average expression of 20 SBF/MBF targets in (A) *ace2* and ACE2*, (B) *ash1* and ASH1*, (C) *ace2 ash1* and ACE2* ASH1* cells. Distribution of cell size at birth after release from the *cdc20* arrest for (D) *ace2 ash1* and (E) ACE2* ASH1* cells.
(1.48 MB TIF)

Figure S4 Deletion of *ACE2* and *ASH1* result in similar T_1_ only in mothers and daughter of similar size. Histogram of the difference in T_1_ for mother-daughter pairs in wild-type (A, B), *ace2* (C, D), *ash1* (E, F), and *ace2 ash1* (G, H) cells. T_1_ is longer in daughters for almost all mother-daughter pairs, indicating that symmetrical regulation of Start is restricted to mothers and daughters of similar size upon deletion of *ACE2* and *ASH1*.
(1.98 MB TIF)

Figure S5 Symmetrical distribution of Ace2 and Ash1 result in similar T_1_ only in mothers and daughter of similar size. Histogram of the difference in T_1_ for mother-daughter pairs in wild-type (A, B), ACE2* (C, D), ASH1* (E, F), and ACE2* ASH1* (G, H) cells. T_1_ is longer in daughters for almost all mother-daughter pairs, indicating that symmetrical regulation of Start is restricted to mothers and daughters of similar size.
(2.07 MB TIF)

Figure S6 Deletion or symmetrical regulation of *CLN3* result in similar T_1_ only in mothers and daughter of similar size. Histogram of the difference in T_1_ for mother-daughter pairs in wild-type (A, B), *cln3* (C, D), *6xCDC28pr-CLN3* (E), *4xCDC28pr-CLN3* (F), and *ADH1pr-CLN3* (G) cells. T_1_ is longer in daughters for almost all mother-daughter pairs, indicating that symmetrical regulation of Start is restricted to mothers and daughters of similar size.
(1.98 MB TIF)

Figure S7 Start control is similar in mothers and “pseudo-mothers.” Plot of αT_1_ versus ln(M_birth_) for the average “mother-like” (red dots and error bars, see Figure 3) compared to mothers and “pseudo-mothers” (black dots).
(1.45 MB TIF)

Figure S8 Start control is similar in daughters and “pseudo-daughters.” Plot of αT_1_ versus ln(M_birth_) for the average “daughter-like” (blue dots and error bars, see Figure 3) compared to daughters and “pseudo-daughters” (black dots).
(1.53 MB TIF)

Figure S9 Correlation between αT_1_ and ln(M_birth_) for cells grown in glucose in mutants lacking the Ace2/Swi5 and/or Ash1 sites on the *CLN3* promoter. (A) wild-type, (B) Ace2/Swi5 sites mutated, (C) Ash1 sites mutated, (D) Ace2/Swi5 and Ash1 sites mutated. Red dots, mothers; blue dots, daughters.
(1.21 MB TIF)

Figure S10 Ace2 nuclear residence is independent of cell size. Correlation between αT_A2_, that is, the time of Ace2 nuclear residence scaled with growth rate α, and ln(M_birth_) for wild-type daughter cells grown in glucose. Red line: least square fit, slope ≈−0.2.
(0.46 MB TIF)

Figure S11 Phylogenetic analysis of Ace2/Swi5 putative binding sites on the *CLN3* promoter. Ace2/Swi5 consensus-binding site identified by PhyloGibbs as over-represented motif in the promoter of the Ace2 and Swi5 targets. Conserved Ace2/Swi5 putative binding sites identified by PhyloGibbs.
(0.72 MB TIF)

Table S1 Strains list.
(0.10 MB PDF)

Table S2 Plasmids list.
(0.06 MB PDF)

Table S3 Analysis of Ace2 and Ash1 shared targets.
(0.06 MB PDF)

Table S4 Average daughter delay in new-born cells of the same size.
(0.07 MB PDF)

Table S5 Size-independent noise is similar in daughters and pseudo-daughters.
(0.06 MB PDF)

Table S6 Mutation of Ace2/Swi5 and Ash1 putative sites results in reduced binding of these factors to the *CLN3* promoter.
(0.07 MB PDF)

Text S1 Supplementary materials and methods and supplementary results.
(0.13 MB PDF)

## Abbreviations

ChIP: chromatin immuno-precipitation
DDE: Daughter Delay Elements
ECB: early cell-cycle box
