# Supplementary figures and images for: Daughter-Specific Transcription Factors Regulate Cell Size Control in Budding Yeast
Source: PLoS Biol. 2009 Oct 20;7(10):e1000221. doi: 10.1371/journal.pbio.1000221 (PMC2756959; doi:10.1371/journal.pbio.1000221)

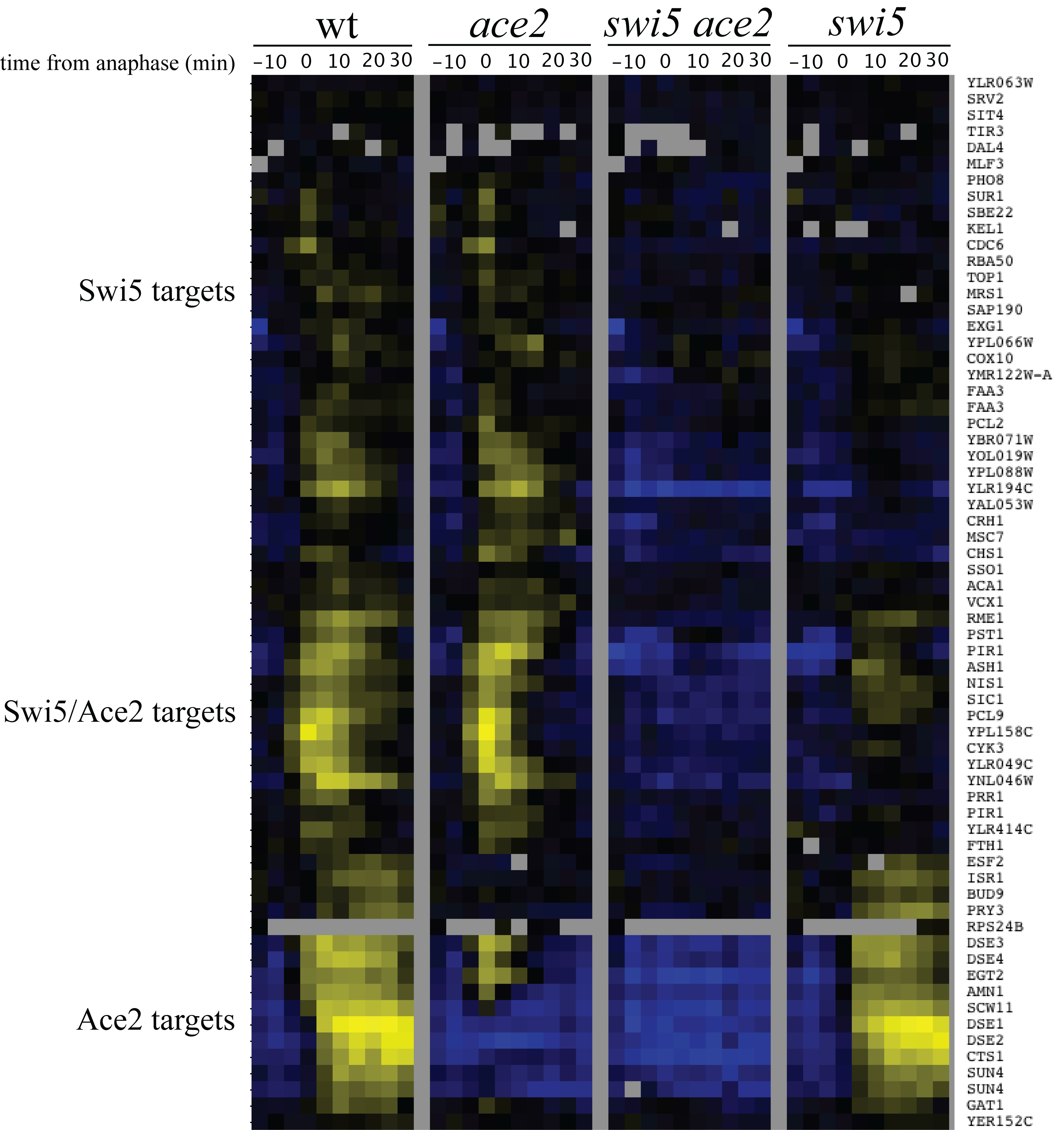

Supplement: Figure S1 — Hierarchical clustering analysis of genes regulated by Ace2 and Swi5. (9.90 MB TIF) [file pbio.1000221.s003.tif]

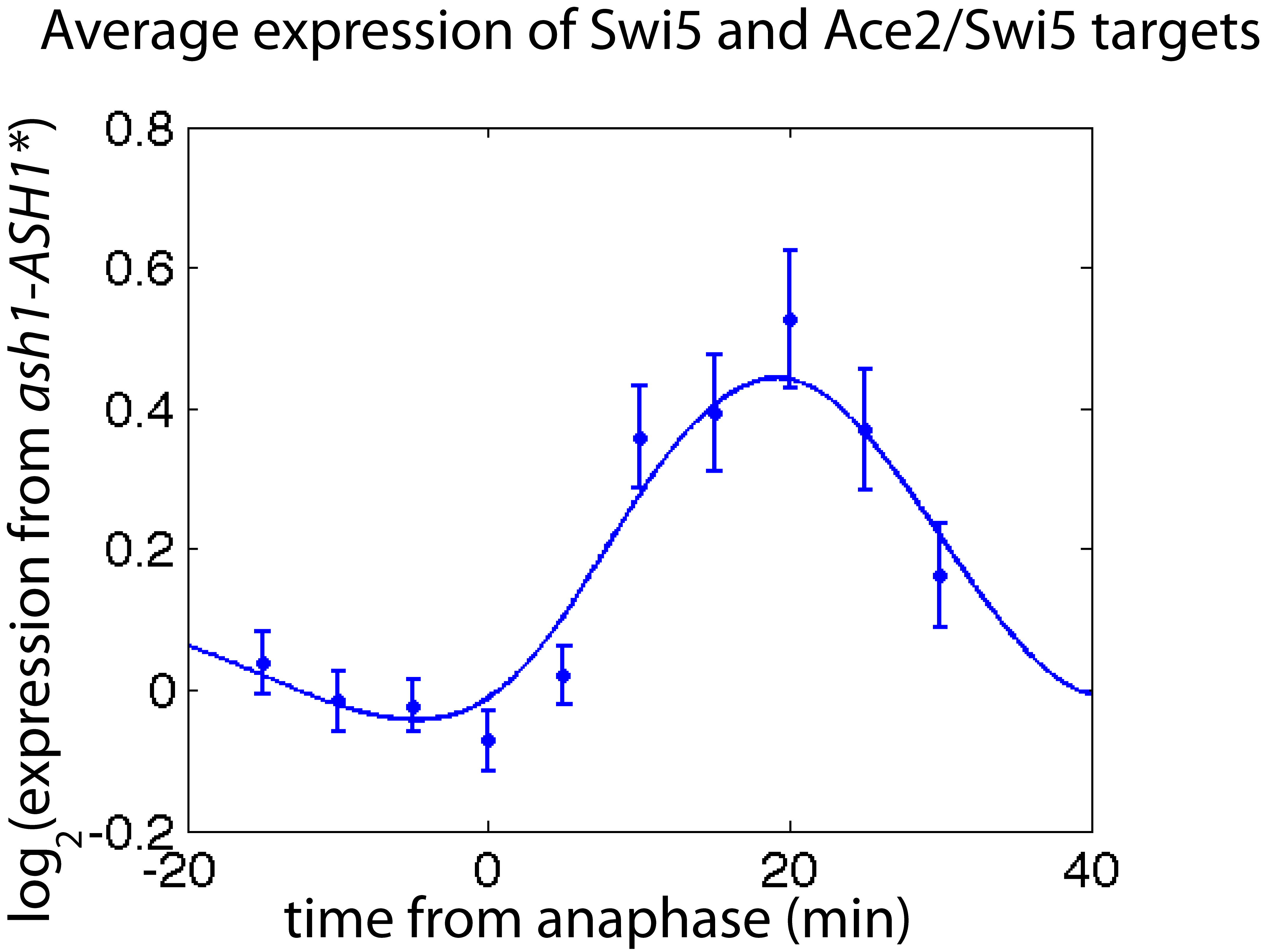

Supplement: Figure S2 — Ash1 is a modulator of Swi5-dependent expression. Average expression for Ace2/Swi5 and Swi5 targets (45 genes) in response to Ash1 (data were obtained by subtracting the ASH1* dataset from ash1 dataset). This graph shows that Ash1 weakly represses the expression of many Ace2/Swi5 and Swi5 targets in daughter cells. (0.87 MB TIF) [file pbio.1000221.s004.tif]

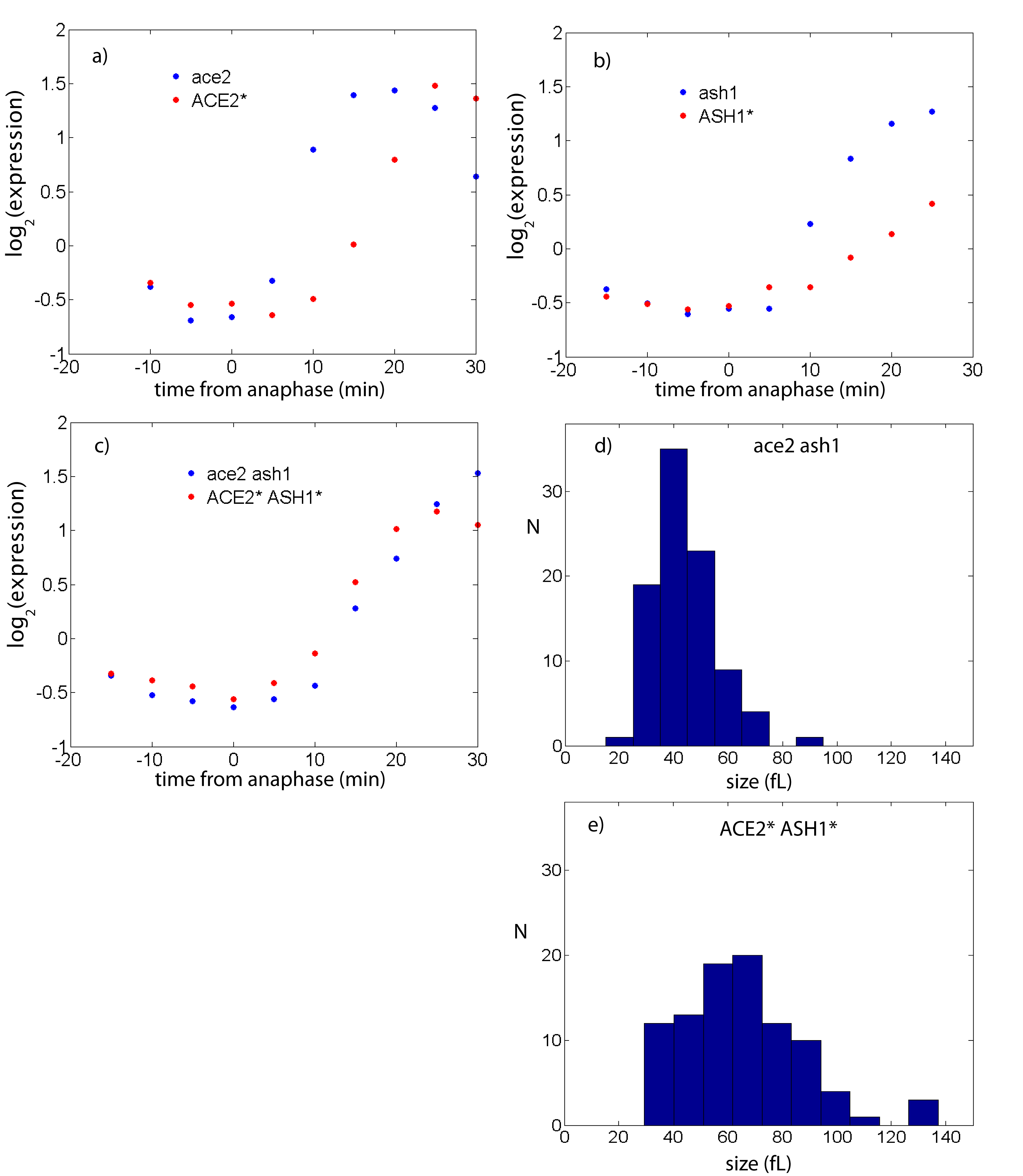

Supplement: Figure S3 — Activation of SBF and MBF is delayed by Ace2 and Ash1. Average expression of 20 SBF/MBF targets in (A) ace2 and ACE2*, (B) ash1 and ASH1*, (C) ace2 ash1 and ACE2* ASH1* cells. Distribution of cell size at birth after release from the cdc20 arrest for (D) ace2 ash1 and (E) ACE2* ASH1* cells. (1.48 MB TIF) [file pbio.1000221.s005.tif]

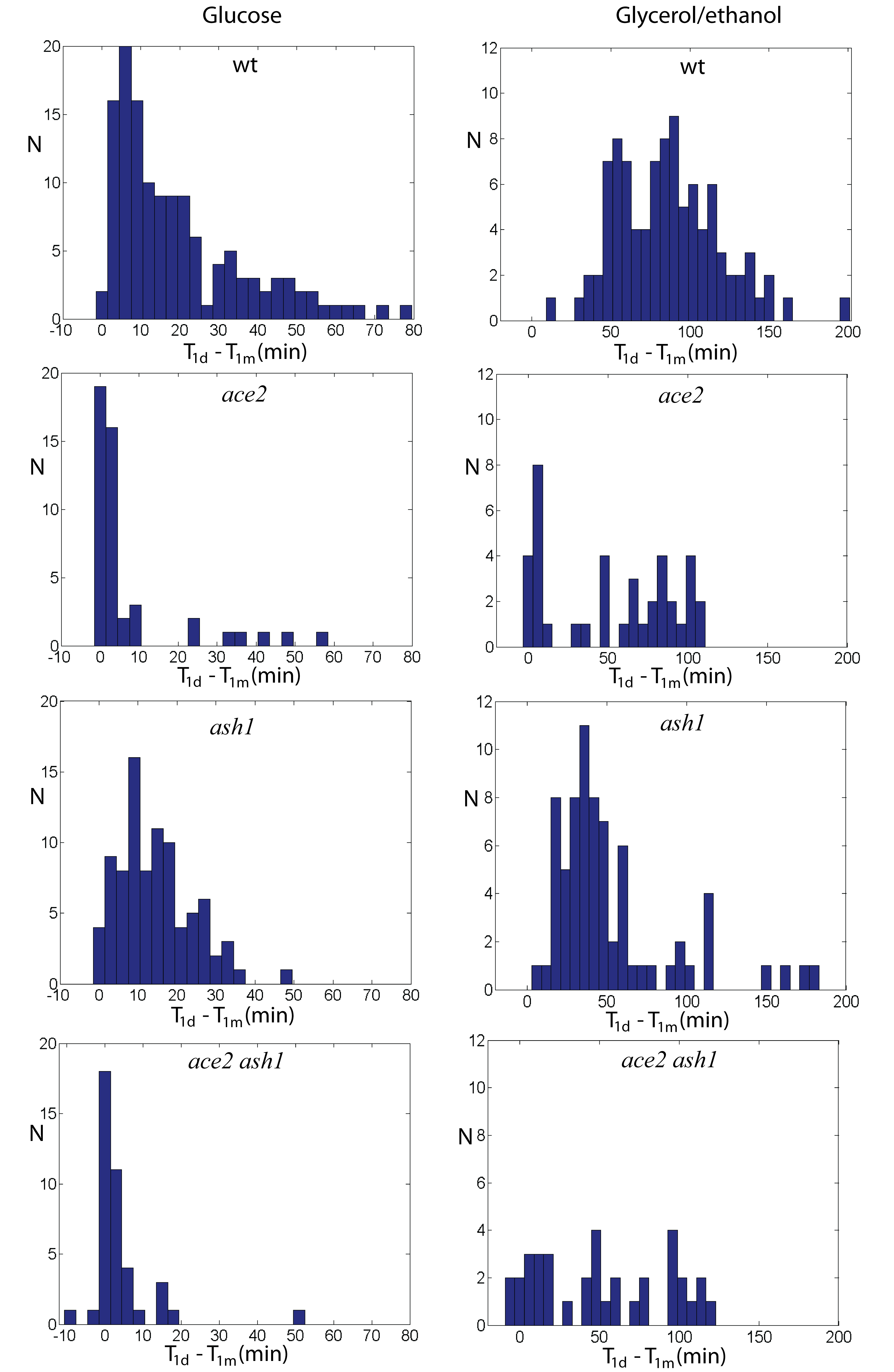

Supplement: Figure S4 — Deletion of ACE2 and ASH1 result in similar T1 only in mothers and daughter of similar size. Histogram of the difference in T1 for mother-daughter pairs in wild-type (A, B), ace2 (C, D), ash1 (E, F), and ace2 ash1 (G, H) cells. T1 is longer in daughters for almost all mother-daughter pairs, indicating that symmetrical regulation of Start is restricted to mothers and daughters of similar size upon deletion of ACE2 and ASH1. (1.98 MB TIF) [file pbio.1000221.s006.tif]

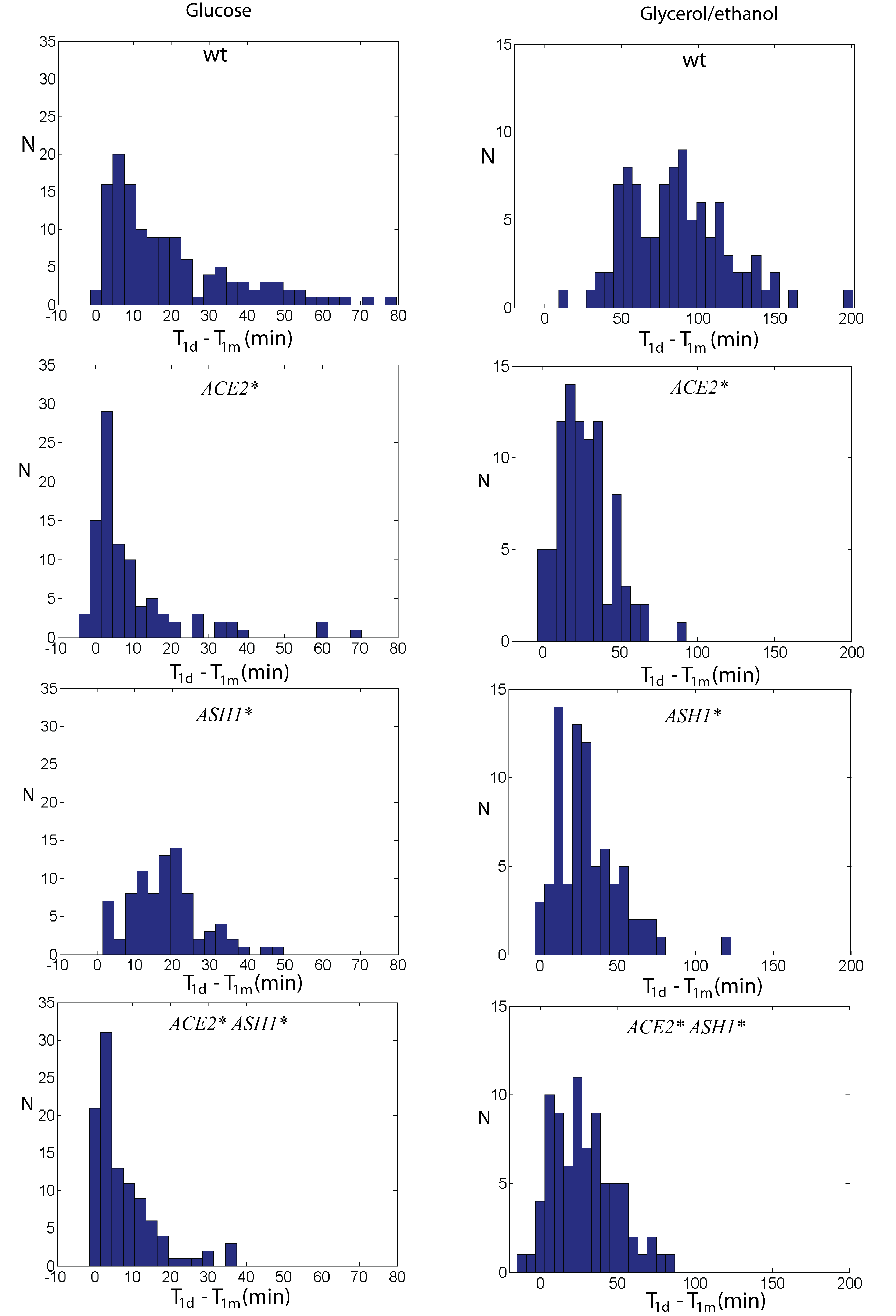

Supplement: Figure S5 — Symmetrical distribution of Ace2 and Ash1 result in similar T1 only in mothers and daughter of similar size. Histogram of the difference in T1 for mother-daughter pairs in wild-type (A, B), ACE2* (C, D), ASH1* (E, F), and ACE2* ASH1* (G, H) cells. T1 is longer in daughters for almost all mother-daughter pairs, indicating that symmetrical regulation of Start is restricted to mothers and daughters of similar size. (2.07 MB TIF) [file pbio.1000221.s007.tif]

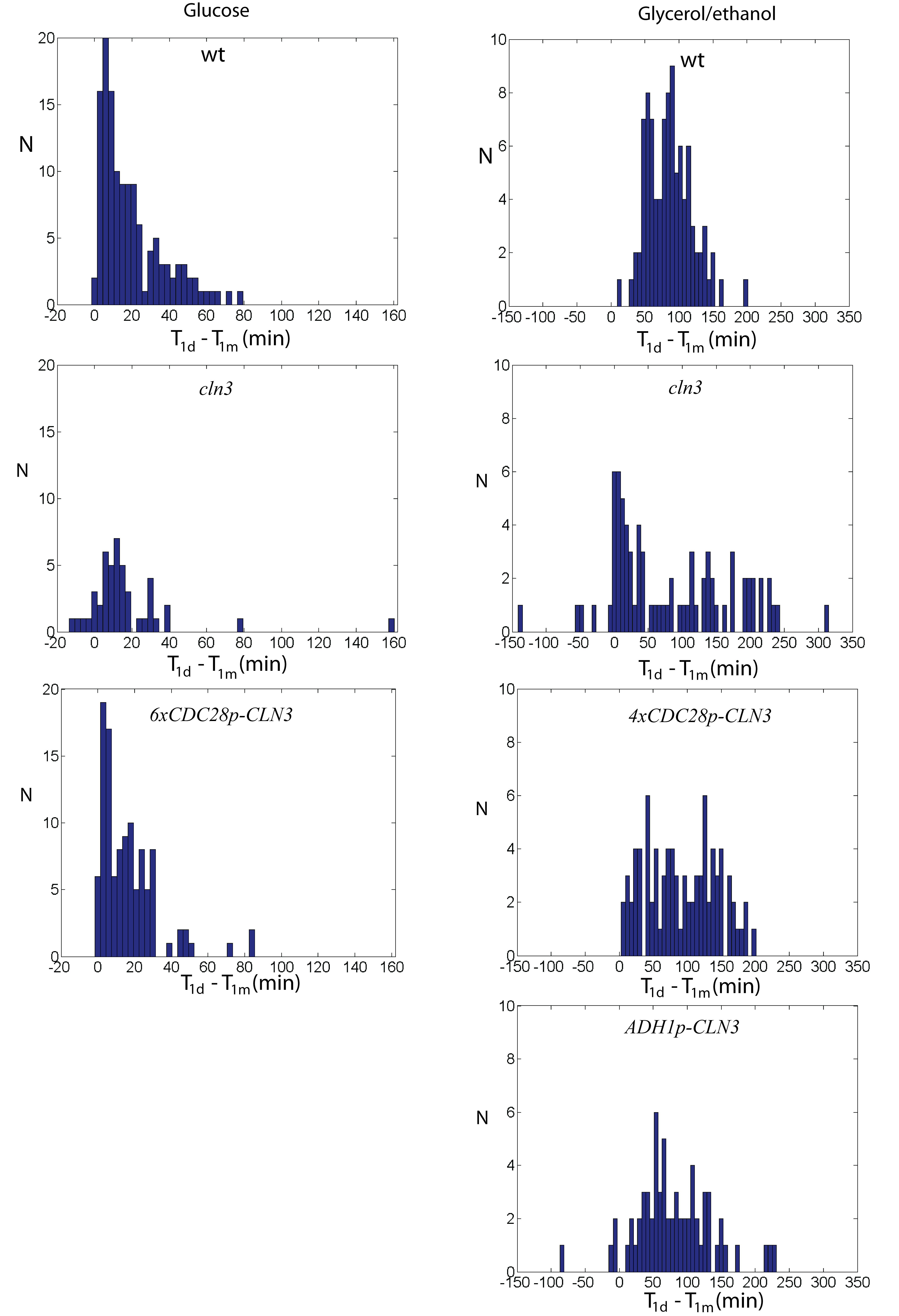

Supplement: Figure S6 — Deletion or symmetrical regulation of CLN3 result in similar T1 only in mothers and daughter of similar size. Histogram of the difference in T1 for mother-daughter pairs in wild-type (A, B), cln3 (C, D), 6xCDC28pr-CLN3 (E), 4xCDC28pr-CLN3 (F), and ADH1pr-CLN3 (G) cells. T1 is longer in daughters for almost all mother-daughter pairs, indicating that symmetrical regulation of Start is restricted to mothers and daughters of similar size. (1.98 MB TIF) [file pbio.1000221.s008.tif]

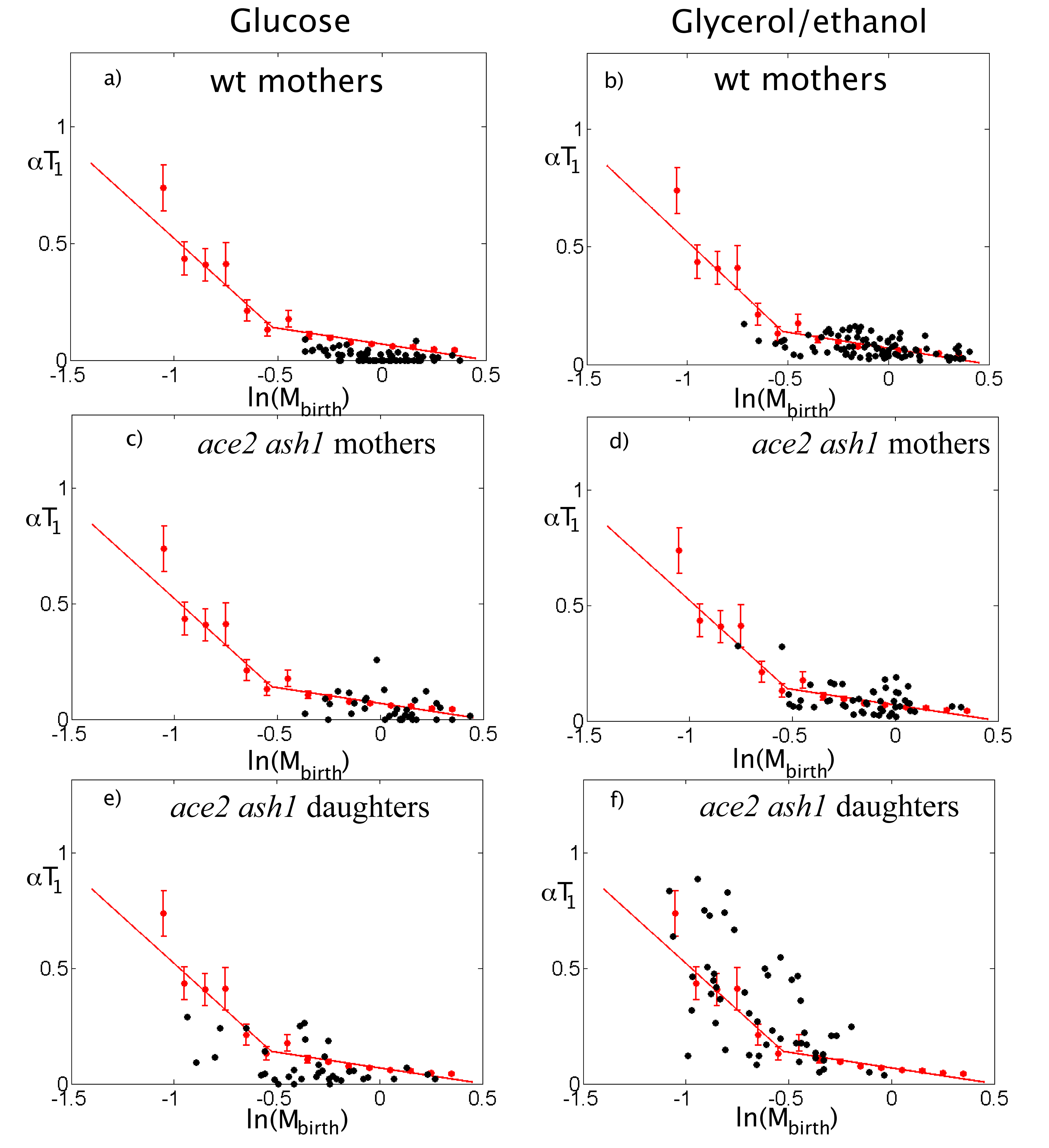

Supplement: Figure S7 — Start control is similar in mothers and “pseudo-mothers.” Plot of αT1 versus ln(Mbirth) for the average “mother-like” (red dots and error bars, see Figure 3) compared to mothers and “pseudo-mothers” (black dots). (1.45 MB TIF) [file pbio.1000221.s009.tif]

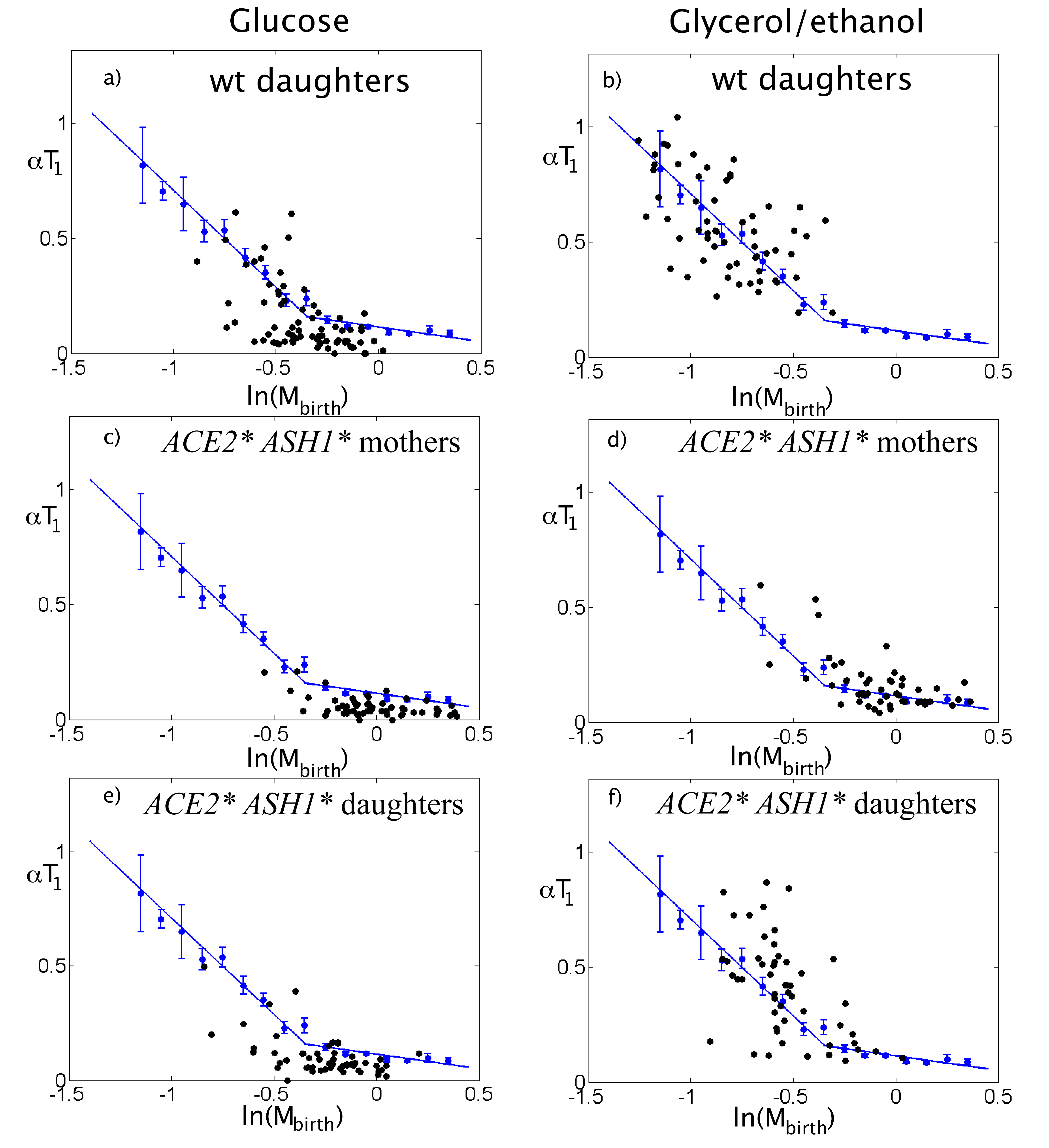

Supplement: Figure S8 — Start control is similar in daughters and “pseudo-daughters.” Plot of αT1 versus ln(Mbirth) for the average “daughter-like” (blue dots and error bars, see Figure 3) compared to daughters and “pseudo-daughters” (black dots). (1.53 MB TIF) [file pbio.1000221.s010.tif]

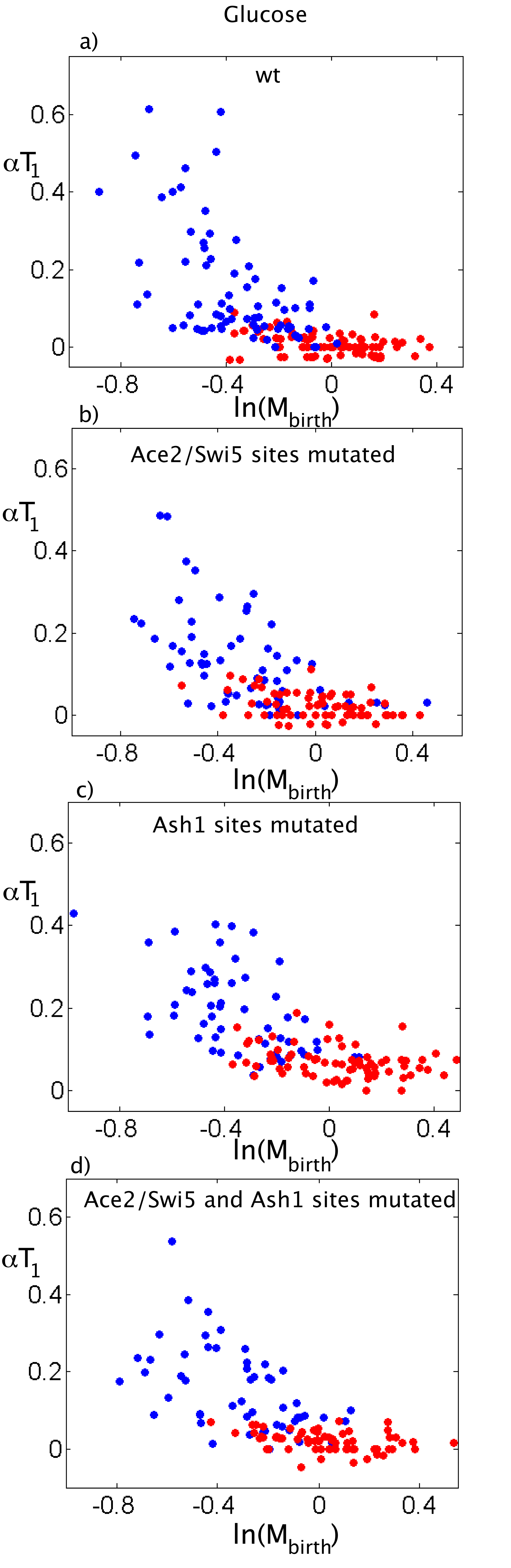

Supplement: Figure S9 — Correlation between αT1 and ln(Mbirth) for cells grown in glucose in mutants lacking the Ace2/Swi5 and/or Ash1 sites on the CLN3 promoter. (A) wild-type, (B) Ace2/Swi5 sites mutated, (C) Ash1 sites mutated, (D) Ace2/Swi5 and Ash1 sites mutated. Red dots, mothers; blue dots, daughters. (1.21 MB TIF) [file pbio.1000221.s011.tif]

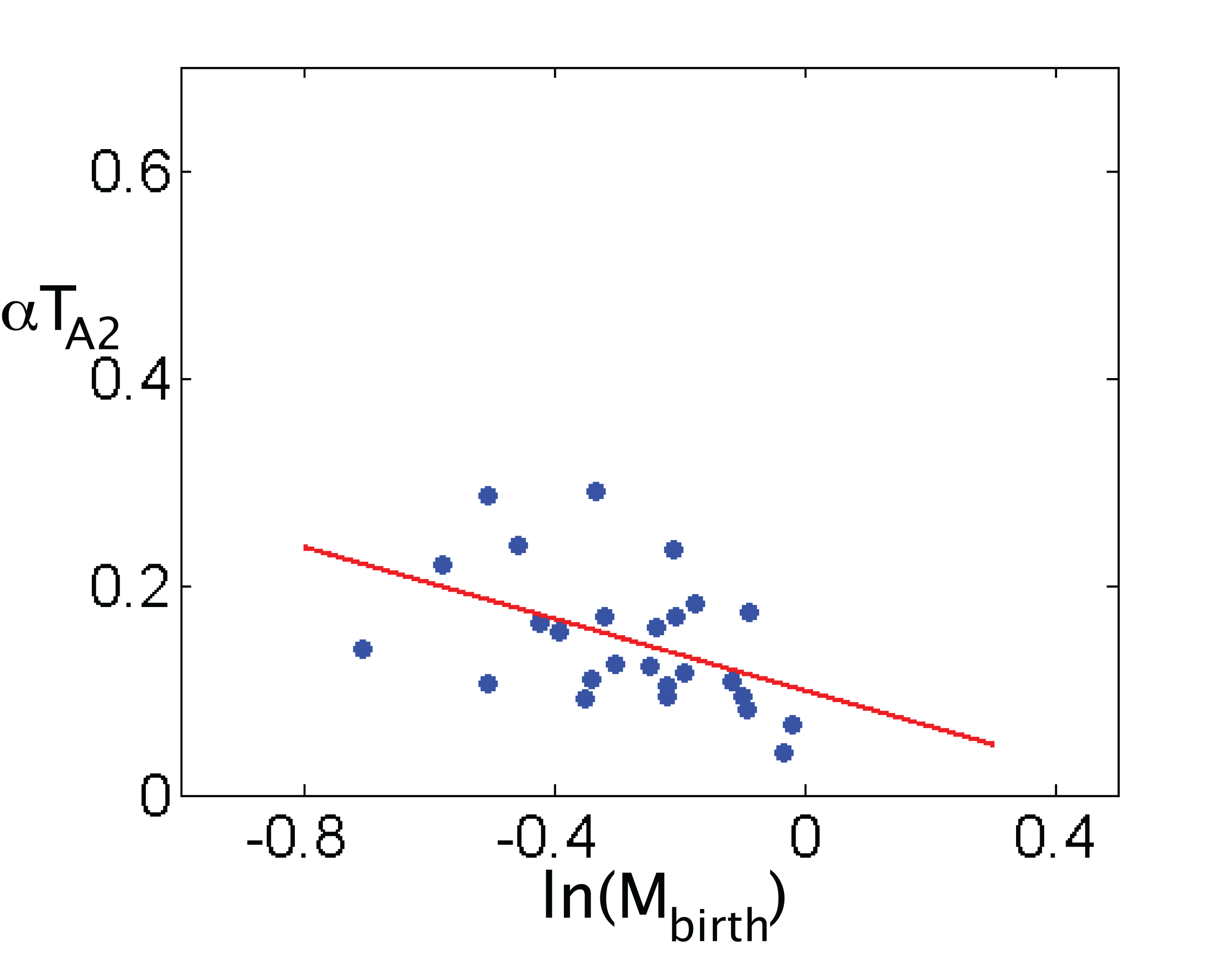

Supplement: Figure S10 — Ace2 nuclear residence is independent of cell size. Correlation between αTA2, that is, the time of Ace2 nuclear residence scaled with growth rate α, and ln(Mbirth) for wild-type daughter cells grown in glucose. Red line: least square fit, slope ≈−0.2. (0.46 MB TIF) [file pbio.1000221.s012.tif]

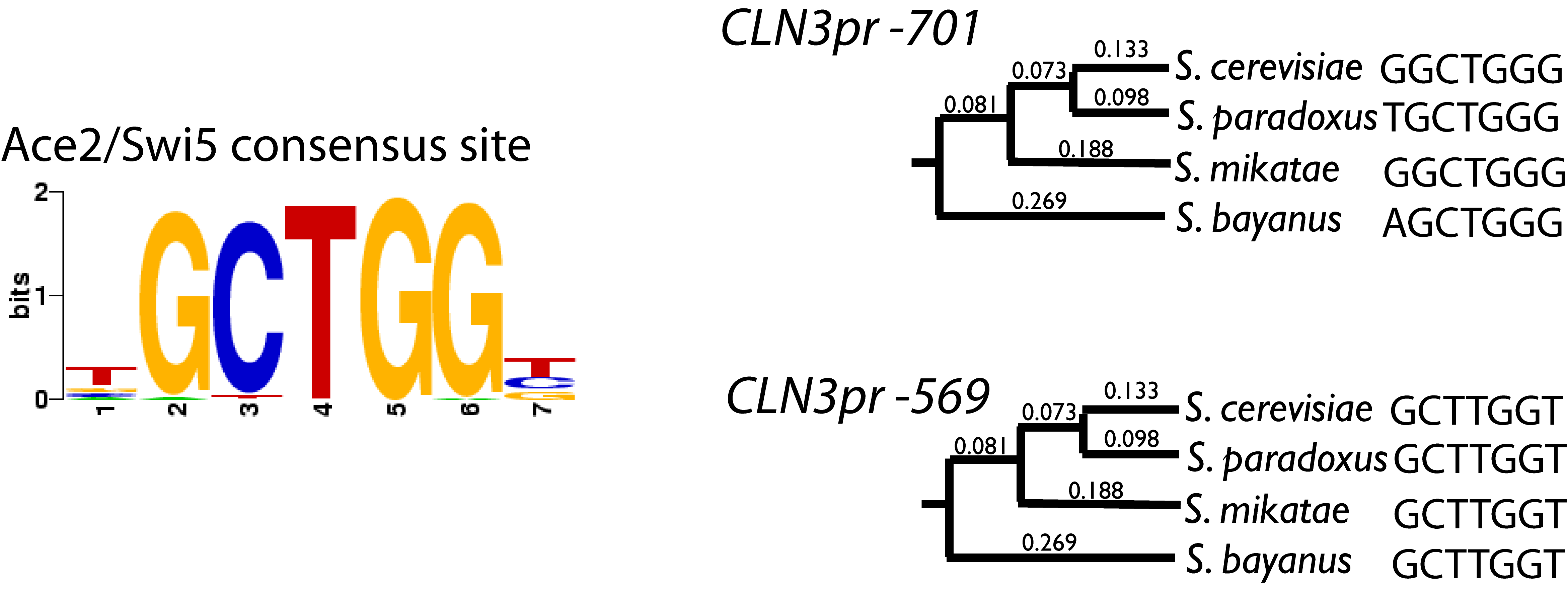

Supplement: Figure S11 — Phylogenetic analysis of Ace2/Swi5 putative binding sites on the CLN3 promoter. Ace2/Swi5 consensus-binding site identified by PhyloGibbs as over-represented motif in the promoter of the Ace2 and Swi5 targets. Conserved Ace2/Swi5 putative binding sites identified by PhyloGibbs. (0.72 MB TIF) [file pbio.1000221.s013.tif]
