# Supplementary material for: Daughter-Specific Transcription Factors Regulate Cell Size Control in Budding Yeast
Source: PLoS Biol. 2009 Oct 20;7(10):e1000221. doi: 10.1371/journal.pbio.1000221 (PMC2756959; doi:10.1371/journal.pbio.1000221)
Supplement: Table S1 — Strains list. (0.10 MB PDF) [file pbio.1000221.s014.pdf]

**Name**	**Genotype**	**Source**
MMY116-2C	MATα *ADE2*	Mary Miller
SD15-6C	MATa *ACT1pr-DSRED::TRP1 WHI5-GFP::KanMX ADE2*	Stefano Di Talia
SD15-8A	MATα *ACT1pr-DSRED::TRP1 WHI5-GFP::KanMX ADE2*	Stefano Di Talia
SD57-2D	MATa *ACT1pr-DSRED::TRP1 WHI5-GFP::KanMX ADE2 ace2::URA3*	This study
SD57-3B	MATa *ACT1pr-DSRED::TRP1 WHI5-GFP::KanMX ADE2 ace2::URA3*	This study
SD57-9B	MATα *ACT1pr-DSRED::TRP1 WHI5-GFP::KanMX ADE2 ace2::URA3*	This study
SD76-1-1C	MATα *ACT1pr-DSRED::TRP1 WHI5-GFP::KanMX ADE2 ash1:: KanMX*	This study
SD76-1-5A	MATa *ACT1pr-DSRED::TRP1 WHI5-GFP::KanMX ADE2 ash1:: KanMX*	This study
SD76-3-12B	MATa *ACT1pr-DSRED::TRP1 WHI5-GFP::KanMX ADE2 ash1:: KanMX*	This study
SD58-5-2A	MATa *ACT1pr-DSRED::TRP1 WHI5-GFP::KanMX ADE2 ash1:: KanMX ace2::URA3*	This study
SD33-1D	MATa *ACT1pr-DSRED::TRP1 WHI5-GFP::KanMX ADE2 ace2::HIS3::ACE2G128E::URA3*	This study
SD33-2A	MATa *ACT1pr-DSRED::TRP1 WHI5-GFP::KanMX ADE2 ace2::HIS3::ACE2G128E::URA3*	This study
SD33-5C	MATa *ACT1pr-DSRED::TRP1 WHI5-GFP::KanMX ADE2 ace2::HIS3::ACE2G128E::URA3*	This study
SD50-13C	MATα *ACT1pr-DSRED::TRP1 WHI5-GFP::KanMX ADE2 ash1:: KanMX ASH1-MUT::LEU2*	This study
SD50-11A	MATa *ACT1pr-DSRED::TRP1 WHI5-GFP::KanMX ADE2 ace2::HIS3::ACE2G128E::URA3 ash1:: KanMX ASH1-MUT::LEU2*	This study
FC2147-7C	MATα *cdc20::LEU2 GALL-CDC20::ADE2*	Frederick Cross
SD59-6C	MATa *cdc20::LEU2 GALL-CDC20::ADE2 ACE2-YFP::URA3*	This study
SD60-4C	MATα *cdc20::LEU2 GALL-CDC20::ADE2 SWI5-GFP::KanMX*	This study
SD62-7C	MATα *cdc20::LEU2 GALL-CDC20::ADE2 ASH1-GFP::KanMX*	This study
SD42-7A	MATα *MYO1-mCherry::HIS5 ACE2-YFP::URA3 ADE2*	This study
SD73-8A	MATa *MYO1-mCherry::HIS5 SWI5-GFP::KanMX ADE2*	This study
SD74-9C	MATa *MYO1-mCherry::HIS5 ASH1-GFP::KanMX ADE2*	This study
JB55-4C	MATα *cdc20::LEU2 GALL-CDC20::ADE2 ace2::URA3*	James Bean
JB55-8A	MATa *cdc20::LEU2 GALL-CDC20::ADE2 swi5::KanMX*	James Bean
JB55-13C	MATα *cdc20::LEU2 GALL-CDC20::ADE2 swi5::KanMX ace2::URA3*	James Bean
SD49-1-1B	MATα *cdc20::LEU2 GALL-CDC20::ADE2 ace2::HIS3::ACE2G128E::URA3*	This study
SD51-10B	MATa *cdc20::LEU2 GALL-CDC20::ADE2 ash1:: KanMX HOpr-CAN1 HOpr-ADE2*	This study
SD51-12B	MATa *cdc20::LEU2 GALL-CDC20::ADE2 ash1:: KanMX ASH1-MUT::LEU2 HOpr-CAN1 HOpr-ADE2*	This study
SD69-2C	MATα *cdc20::LEU2 GALL-CDC20::ADE2 ash1:: KanMX HOpr-CAN1 ace2::URA3*	This study
SD70-12B	MATα *cdc20::LEU2 GALL-CDC20::ADE2 ash1:: KanMX ASH1-MUT::LEU2 ace2::HIS3::ACE2G128E::URA3 HOpr-CAN1*	This study
SD52-2A	MATa *cdc20::LEU2 GALL-CDC20::ADE2 ACE2-TAP::HIS3*	This study
SD53-3B	MATα *cdc20::LEU2 GALL-CDC20::ADE2 SWI5-TAP::HIS3*	This study
SD72-9C	MATa *cdc20::LEU2 GALL-CDC20::ADE2 ASH1-TAP::HIS3*	This study

SD28-3C	MATa *ACT1pr-DSRED::TRP1WHI5-GFP::KanMX cln3::URA3 ADE2*	Stefano Di Talia
SD28-5A	MATα *ACT1pr-DSRED::TRP1 WHI5-GFP::KanMX cln3::URA3 ADE2*	Stefano Di Talia
SD65-1	MATa *ACT1pr-DSRED::TRP1 WHI5-GFP::KanMX CLN3-9xMYC::HIS3 ADE2*	This study
SD65-2	MATa *ACT1pr-DSRED::TRP1 WHI5-GFP::KanMX CLN3-9xMYC::HIS3 ADE2*	This study
SD66-1	MATa *ACT1pr-DSRED::TRP1 WHI5-GFP::KanMX ACT1pr-CLN3-9xMYC::HIS3::LEU2 ADE2*	This study
SD66-2	MATa *ACT1pr-DSRED::TRP1 WHI5-GFP::KanMX ACT1pr-CLN3-9xMYC::HIS3::LEU2 ADE2*	This study
SD67-1	MATa *ACT1pr-DSRED::TRP1 WHI5-GFP::KanMX ADH1pr-CLN3-9xMYC::HIS3::LEU2 ADE2*	This study
SD67-2	MATa *ACT1pr-DSRED::TRP1 WHI5-GFP::KanMX ADH1pr-CLN3-9xMYC::HIS3::LEU2 ADE2*	This study
SD71-1	MATa *ACT1pr-DSRED::TRP1 WHI5-GFP::KanMX CDC28pr-CLN3-9xMYC::HIS3::LEU2 ADE2*	This study
SD71-2	MATa *ACT1pr-DSRED::TRP1 WHI5-GFP::KanMX CDC28pr-CLN3-9xMYC::HIS3::LEU2 ADE2*	This study
SD75-1	MATα *ACT1pr-DSRED::TRP1 WHI5-GFP::KanMX cln3::URA3 CDC28pr-CLN3::LEU2 5xCDC28pr-CLN3::HIS3 ADE2*	This study
SD75-3	MATα *ACT1pr-DSRED::TRP1 WHI5-GFP::KanMX cln3::URA3 CDC28pr-CLN3::LEU2 3xCDC28pr-CLN3::HIS3 ADE2*	This study
SD54-1	MATα *ACT1pr-DSRED::TRP1 WHI5-GFP::KanMX cln3::URA3 ADH1pr-CLN3::LEU2 ADE2*	This study
SD54-2	MATα *ACT1pr-DSRED::TRP1 WHI5-GFP::KanMX cln3::URA3 ADH1pr-CLN3::LEU2 ADE2*	This study
SD80-1	MATa *ACT1pr-DSRED::TRP1 WHI5-GFP::KanMX ADE2 Ace2/Swi5 binding sites mutated on the CLN3 promoter*	This study
SD80-2	MATα *ACT1pr-DSRED::TRP1 WHI5-GFP::KanMX ADE2 Ace2/Swi5 binding sites mutated on the CLN3 promoter*	This study
SD81-1	MATa *ACT1pr-DSRED::TRP1 WHI5-GFP::KanMX ADE2 Ash1 binding sites mutated on the CLN3 promoter*	This study
SD81-2	MATα *ACT1pr-DSRED::TRP1 WHI5-GFP::KanMX ADE2 Ash1 binding sites mutated on the CLN3 promoter*	This study
SD82-1	MATa *ACT1pr-DSRED::TRP1 WHI5-GFP::KanMX ADE2 Ace2/Swi5 and Ash1 binding sites mutated on the CLN3 promoter*	This study
SD82-2	MATα *ACT1pr-DSRED::TRP1 WHI5-GFP::KanMX ADE2 Ace2/Swi5 and Ash1 binding sites mutated on the CLN3 promoter*	This study
SD83-1	MATa *ACT1pr-DSRED::TRP1 WHI5-GFP::KanMX ADE2 CLN3::URA3*	This study
SD83-2	MATα *ACT1pr-DSRED::TRP1 WHI5-GFP::KanMX ADE2 CLN3::URA3*	This study
SD37-2A	*ACT1pr-DSRED::TRP1 ACE2-YFP::URA3 ADE2*	This study
SD88-3-2B	*MET3pr-CLN2::TRP1 ACE2-YFP::URA3 MYO1-mCherry::HIS5 ADE2*	This study
SD88-1-3B	*MET3pr-CLN2::TRP1 ACE2-YFP::URA3 MYO1-mCherry::HIS5 cln3::URA3 ADE2*	This study
HWL182	MATa/MATα *ACE2-TAP::HIS3/ACE2-TAP::HIS3 wild-type CLN3 promoter/CLN3 promoter with Ace2/Swi5 and Ash1 binding sites mutated*	This study
HWL183	MATa/MATα *ASH1-TAP::HIS3/ASH1-TAP::HIS3 wild-type CLN3 promoter/CLN3 promoter with Ace2/Swi5 and Ash1 binding sites mutated*	This study

HWL184	MATa/MATα *SWI5-TAP::HIS3/SWI5-TAP::HIS3 wild-type CLN3 promoter/CLN3 promoter with Ace2/Swi5 and Ash1 binding sites mutated*	This study

Table S1 Strains list All strains are congenic W303 (*leu2-3,112 his3-11,15 ura3-1 trp1-1 can1-1*) and were constructed from lab stocks by standard methods. All the strains used in time-lapse microscopy experiments are *ADE2*. The *ADE2* conversion was used to reduce vacuolar autofluorescence that can occur in *ade2* strains.
