# Supplementary material for: Daughter-Specific Transcription Factors Regulate Cell Size Control in Budding Yeast
Source: PLoS Biol. 2009 Oct 20;7(10):e1000221. doi: 10.1371/journal.pbio.1000221 (PMC2756959; doi:10.1371/journal.pbio.1000221)
Supplement: Table S2 — Plasmids list. (0.06 MB PDF) [file pbio.1000221.s015.pdf]

Name	Description	Construction
p405CYC1	pRS405-*CYC1pr*	Nicolas Buchler
pSD07	pRS405-*CYC1pr-CLN3*	see above
pSD08	pRS405-*CDC28pr-CLN3*	see above
pSD09	pRS405-*ACT1pr-CLN3*	see above
p405ADH1	pRS405-*ADH1pr*	Nicolas Buchler
pSD10	pRS405-*ADH1pr-CLN3*	see above
pSD13	pRS403-*CDC28pr-CLN3*	see above
pMM99	pRS414-*CLN3-9xMYC*	Mary Miller
pSD14	pRS403-truncated *CLN3-9xMYC*	see above
FC101	YCp50 without centromere +6.5 Kb *CLN3* genomic region	Fred Cross
pSD15	FC101 with Ace2/Swi5 sites on the *CLN3* promoter mutated	see above
pSD16	FC101 with Ash1 sites on the *CLN3* promoter mutated	see above
pSD17	FC101 with Ace2/Swi5 and Ash1 sites on the *CLN3* promoter mutated	see above

Table S2 Plasmids list
