# Supplementary material for: Daughter-Specific Transcription Factors Regulate Cell Size Control in Budding Yeast
Source: PLoS Biol. 2009 Oct 20;7(10):e1000221. doi: 10.1371/journal.pbio.1000221 (PMC2756959; doi:10.1371/journal.pbio.1000221)
Supplement: Table S3 — Analysis of Ace2 and Ash1 shared targets. (0.06 MB PDF) [file pbio.1000221.s016.pdf]

Gene name	p value in *ACE2**-*ace2* dataset	p value in *ASH1**-*ash1* dataset	p value in *ASH1* ACE2**-*ash1 ace2* dataset (see below)
CLN3	0.02	$4*10^{-4}$	0.04
HSP150	0.001	0.01	0.03
MET6	$4*10^{-6}$	0.01	0.04
YRF1-1	0.02	$2*10^{-4}$	0.04
YRF1-5	0.0068	$3*10^{-5}$	0.03

Table S3 Analysis of Ace2 and Ash1 shared targets. We report the p-values that the expression of listed genes is not affected by Ace2 and Ash1. For unknown experimental reasons the error-bars on the *ASH1* ACE2*-ash1 ace2* dataset were on average two times larger than the error-bars on the *ACE2*-ace2* and *ASH1*-ash1* datasets.
