# Supplementary material for: Daughter-Specific Transcription Factors Regulate Cell Size Control in Budding Yeast
Source: PLoS Biol. 2009 Oct 20;7(10):e1000221. doi: 10.1371/journal.pbio.1000221 (PMC2756959; doi:10.1371/journal.pbio.1000221)
Supplement: Table S4 — Average daughter delay in new-born cells of the same size. (0.07 MB PDF) [file pbio.1000221.s017.pdf]

	wt	*ace2*	*ash1*	*ace2 ash1*
Daughter-mother delay in glucose	8 ± 1 min (10^{-6})	2 ± 3 min (0.31)	6 ± 1 min (10^{-5})	3 ± 2 min (0.69)
Daughter-mother delay in gly/eth	87 ± 9 min ($<10^{-70}$)	16 ± 13 min (0.54)	40 ± 8 min (10^{-8})	17 ± 9 min (0.05)
	**wt**	***ACE2****	***ASH1****	***ASH1* ACE2****
Daughter-mother delay in glucose	8 ± 1 min (10^{-6})	1.3 ± 0.9 min (0.12)	5 ± 1 min (10^{-5})	1.3 ± 0.9 min (0.06)
Daughter-mother delay in gly/eth	87 ± 9 min ($<10^{-70}$)	37 ± 12 min (10^{-5})	19 ± 7 min (0.04)	5 ± 7 min (0.41)
	**wt**	**Ace2/Swi5 sites mutated**	**Ash1 sites mutated**	**Ace2/Swi5 and Ash1 sites mutated**
Daughter-mother delay in glucose	8 ± 1 min (10^{-6})	8 ± 2 min (10^{-5})	10 ± 2 min (10^{-4})	7 ± 1 min (10^{-5})
Daughter-mother delay in gly/eth	87 ± 9 min ($<10^{-70}$)	46 ± 7 min (10^{-18})	47 ± 15 min (10^{-15})	54 ± 9 min (10^{-19})
	**wt**	***cln3***	***ADH1pr-CLN3***	***nxCDC28pr-CLN3***
Daughter-mother delay in glucose	8 ± 1 min (10^{-6})	3 ± 1 min (0.04)	N/A	3 ± 1 min (0.19)
Daughter-mother delay in gly/eth	87 ± 9 min ($<10^{-70}$)	9 ± 13 min (0.22)	22 ± 10 min (0.37)	33 ± 12 min (0.01)

Table S4 Average daughter delay in new-born cells of the same size. Data from the correlation of αT_1 and $\ln(M_{birth})$ were divided in small bins and the daughter delays computed for every bin were averaged. In parenthesis is the p-value -computed via a χ^2 test- for the null hypothesis that mothers and daughters display symmetrical regulation of Start, i.e. the same correlation between αT_1 and $\ln(M_{birth})$.
