# Supplementary material for: Daughter-Specific Transcription Factors Regulate Cell Size Control in Budding Yeast
Source: PLoS Biol. 2009 Oct 20;7(10):e1000221. doi: 10.1371/journal.pbio.1000221 (PMC2756959; doi:10.1371/journal.pbio.1000221)
Supplement: Table S5 — Size-independent noise is similar in daughters and pseudo-daughters. (0.06 MB PDF) [file pbio.1000221.s018.pdf]

	**wt**	***ASH1*ACE2**** **mothers**	***ASH1* ACE2**** **daughters**
Size-independent noise in D	0.79 ± 0.10	0.89 ± 0.16	0.65 ± 0.09
Size-independent noise in gly/eth	0.57 ± 0.08	0.48 ± 0.08	0.45 ± 0.08

Table S5 Size-independent noise is similar in daughters and pseudo-daughters. Size-independent noise was estimated as the error about the binned data as described in Di Talia et al. (2007) Nature, *448*, 947-51. Similar analysis cannot be performed for mothers and pseudo-mothers because the interval T_1 is on average so short that the variability is dominated by the measurement error.
