# Supplementary material for: Daughter-Specific Transcription Factors Regulate Cell Size Control in Budding Yeast
Source: PLoS Biol. 2009 Oct 20;7(10):e1000221. doi: 10.1371/journal.pbio.1000221 (PMC2756959; doi:10.1371/journal.pbio.1000221)
Supplement: Table S6 — Mutation of Ace2/Swi5 and Ash1 putative sites results in reduced binding of these factors to the CLN3 promoter. (0.07 MB PDF) [file pbio.1000221.s019.pdf]

Mutation (position)	**Ace2 ChIP**	**Ash1 ChIP**	**Swi5 ChIP**
C/T (-1183)	0.65	0.54	0.74
G/A (-1181)	0.69	0.57	0.80
C/A (-1124)	0.69	0.67	0.70
C/T (-1015)	0.72	0.64	0.73
G/A (-1013)	0.66	0.59	0.80
C/T (-1000)	0.63	0.55	0.65
T/C (-999)	0.65	0.60	0.73
G/A (-998)	0.64	0.63	0.79
T/C (-767)	1.25	1.28	1.20
G/A (-765)	1.28	1.00	1.20
C/T (-700)	1.19	0.85	0.93
G/A (-698)	1.26	1.12	1.27
C/A (-590)	0.87	0.91	1.02
C/A (-545)	1.06	0.91	1.27

Table S6 Mutation of Ace2/Swi5 and Ash1 putative binding sites results in reduced binding of these factors to the *CLN3* promoter. Binding of Ace2, Ash1 and Swi5 to sequences between -1183 and -998 (ATG:+1) is reduced to about 60% of its wt value, while binding to sequences between -767 and -545 is not affected. The residual signal from the mutant is consistent with either a low level of background precipitation, or to genuine residual binding of the factors to non-consensus sites in the promoter. We observe that background precipitation would make the estimated reduction in binding smaller. This table reports the binding strength to mutated sequences on the *CLN3* promoter relative to the binding to wt sequences. The ratio of mutated sequences to wt sequence for various PCR products after Ace2, Ash1 and Swi5 ChIP was evaluated and normalized by the ratio of mutated sequences to wt sequences for the same PCR products of genomic DNA (sequenced with the same primer). This normalization ensures that the effects are specific to Ace2, Ash1 and Swi5 ChIP and not an artifact of PCR or sequencing.
