# Supplementary material for: Daughter-Specific Transcription Factors Regulate Cell Size Control in Budding Yeast
Source: PLoS Biol. 2009 Oct 20;7(10):e1000221. doi: 10.1371/journal.pbio.1000221 (PMC2756959; doi:10.1371/journal.pbio.1000221)
Supplement: Text S1 — Supplementary materials and methods and supplementary results. (0.13 MB PDF) [file pbio.1000221.s020.pdf]

Text S1

Supplementary Materials and Methods

Plasmids and strains construction.

Plasmid pSD07 was constructed by inserting the *CLN3* ORF (amplified from yeast genome by PCR) in the pRS405-*CYC1pr* (kind gift of Nicolas Buchler) by XbaI and SalI digestion. Plasmids pSD08, pSD09 and pSD10 were constructed by replacing the *CYC1pr* with the *CDC28pr, ACT1pr* and *ADH1pr*. *ACT1pr* and *CDC28pr* were obtained by PCR amplification of yeast genome, while *ADH1pr* was obtained from pRS405-*ADH1pr* (kind gift of Nicolas Buchler). Plasmid pSD13 was obtained from pSD08 by substituting the BglI fragment containing the *LEU2* gene with the BglI fragment containing the *HIS3* gene. Plasmid pSD14 was built from plasmid pMM99 (pRS414-*CLN3-9xMYC*, kind gift of Mary Miller) by replacing the BglI fragment containing the *TRP1* gene with the BglI fragment containing the *HIS3* gene, followed by SalI and SwaI digestion and blunt end ligation. Plasmid pSD15 was obtained by subcloning a fragment containing the mutated *CLN3* promoter and part of *CLN3* ORF from plasmid pBF1-5 in plasmid FC101. Mutations of the Ash1 binding sites on the *CLN3* promoter were introduced by PCR splice overlap of FC101 or pSD15. The mutated *CLN3* promoters obtained from the PCR reactions were inserted into the plasmids pSD16 and pSD17 by XbaI and BclI digestion.

All the strains expressing *CLN3-9xMYC* were generated by transforming in yeast plasmid pSD14 after EcoRI digestion. These strains carry *CLN3-9xMYC* and a truncated not functional *CLN3*. Strains carrying mutations of the Ace2/Swi5 and Ash1 binding sites were generated by transforming in strains SD15-8A and SD15-6C plasmids pSD15, pSD16 and pSD17 after HpaI digestion. Recombinants at the *CLN3* locus were selected on 5'-FOA plates and the *CLN3* promoters amplified by PCR were analyzed by sequencing.

Mutations of the Ace2/Swi5 and Ash1 putative binding sites on the *CLN3* promoter.

We identified in the *CLN3* promoter 3 putative Ace2/Swi5 binding and 2 sites that are over-represented in Ace2 and Swi5 regulated genes (data not shown). We also found 8

putative Ash1 binding sites on the same promoter. We introduced the following mutations of the Ace2/Swi5 putative binding sites (ATG +1): GCCAGCG mutated to GCtAaCG (-1183), TGCTGGC mutated to TGtTaGC (-1016), GGCTGAC mutated to GGtcaAC (-1001), TGCTGAT mutated to TGtTaAT (-766), CCCAGCC mutated to CCtAaCC (-701). We introduced the following mutations of the Ash1 putative binding sites (ATG +1): ATCAA mutated to ATaAA (-1124), CTGAT mutated to CTtAT (-969), CTGAT mutated to tTaAT (-764), ATCAG mutated to ATaAG (-591), ATCAA mutated to ATaAA (-546), TTGAT mutated to TTtAT (-350), CTGAT mutated to CTtAT (-23), TTGAT mutated to TTtAT (-18).

Chromatin immunoprecipitations

Chromatin immunoprecipitations (ChIP) were performed as follows. Early log phase cells were collected and formaldehyde was added to 1% final concentration. Cells were fixed at room temperature for 15 min. Cross-linking was quenched by the addition of glycine to 125 mM. Cells were pelleted at 3,000 rpm for 5 min and washed twice with ice-cold TBS (150 mM NaCl, 20 mM Tris-HCl; pH 7.6). To break cells, cell suspensions in lysis buffer(50mM HEPES-KOH pH7.5, 140mM NaCl, 1mM EDTA, 1% Triton X-100, 0.1% Sodium Deoxycholate) were mixed with glass beads and vortexed at 4°C for 45min. Chromatin was sheared by sonication at power 3(W-380 Sonicator, Heat Systems-Ulrasonic, INC) for 10 times, 10 second each time, and tubes were kept on ice throughout sonication. Cell debris was removed by maximal speed centrifuge for 15min at 4°C. Whole-cell extracts were prepared for use in ChIPs. Protein concentration for each sample was determined by DC protein assay (Bio-Rad).

Immunoprecipitations were performed with 1 mg of extract. Lysates were rotated with 25µl IgG Sepharose beads at 4°C for overnight. Immune complex beads were washed with lysis buffer, lysis buffer 500(50mM HEPES-KOH pH7.5, 500mM NaCl, 1mM EDTA, 1% Triton X-100, 0.1% Sodium Deoxycholate), and LiCl/detergent(0.5% Sodium deoxycholate, 1mMEDTA, 250mM LiCl, 0.5% NP-40, 10mM Tris, pH8.0) twice for each buffer and washed once with cold TE. Beads washing were performed at 4°C. DNA were eluted by incubating beads at 65°C with elution buffer (10mM EDTA, 1%SDS, 50mM Tris.Cl pH8.0) for 10min, and crosslink were reversed by incubating

samples at 65°C overnight. PCR were carried out for 30 cycles and products were viewed with 2.4% agarose gel.

PCR analysis of ChIP experiments.

The following regions of the *DYN1* ORF and the *SIC1*, *CTS1* and *CLN3* promoters were amplified in the PCR reactions reported in Figure 5: *DYN1* (+6091, +6460), *SIC1* (-380, -40), *CTS1* (-658, -400), *CLN3* (-1280 to -964), (-1090 to -890), (-848 to -618), (-432 to –179).

Supplementary results

Analysis of the nuclear localization of Ace2, Ash1 and Swi5 by time-lapse microscopy.

We performed time-lapse microscopy of strains co-expressing Myo1-mCherry and one among Ace2-YFP, Ash1-GFP and Swi5-GFP. Ace2 enters the nucleus of daughters (often a quick translocation through the mother nucleus is also observed) about 8 minutes before cytokinesis (8.4 ± 0.2 min, average ± s.e.m.) and leaves about 15 minutes after cytokinesis (14.6 ± 0.7 min, average ± s.e.m.). Swi5 enters the nucleus of both mother and daughter about 11 minutes before cytokinesis (11.4 ± 0.4 min, average ± s.e.m.) and leaves (or probably is just degraded) few minutes before cytokinesis (4.1 ± 0.5 min, average ± s.e.m.). Ash1 enters the nucleus of daughter cells about 3 minutes before cytokinesis (3.0 ± 0.3 min, average ± s.e.m.) and leaves the nucleus late in G1 or after the cells have budded.

Ace2 nuclear residence is independent of cell size and Cln3.

Ace2 exits the daughter nucleus in G1 often prior to Start. To establish if Ace2 nuclear residence is affected by cell size we analyzed the correlation between αT_{A2}, i.e. the time of Ace2 nuclear residence scaled with growth rate α, and ln (M_{birth}). This analysis demonstrates only a very weak correlation between cell size and Ace2 nuclear residence (slope ≈ -0.2, Figure S10). To analyze if Ace2 nuclear residence is affected by Cln3 activity we measured the time of Ace2 nuclear residence in wt and *cln3* daughter cells

with similar initial size distribution obtained by constitutive expression of *CLN2* from the inducible promoter *MET3pr*. Cells were first grown in medium lacking methionine to express *CLN2* and then switched to medium containing methionine (resulting in *CLN2* expression shut-off). The Ace2 nuclear residence in the first cell cycle after media shift was quantified. This analysis shows that Ace2 nuclear residence is very similar in wt (20 ± 2 min, average ± s.e.m.) and in *cln3* daughters (17 ± 1 min, average ± s.e.m). Together, these results indicate that Ace2 nuclear residence is independent of cell size and Cln3.

Ace2, Swi5 and Ash1 targets from gene array data.

In this Section, we describe the analysis of the microarray data to obtain a list of shared Ace2 and Ash1 targets and the lists of genes whose expression strongly depends on Ace2, Swi5 and Ash1.

First, we did a comprehensive identification of the transcriptioinal targets of Swi5 and Ace2. Because these are cell-cycle transcription factors, the experiments were done in a cell cycle context: a *cdc20 GAL-CDC20* strain was used so that cells could be arrested prior to mitosis, then released into a synchronous cell cycle. In this *cdc20 GAL-CDC20* background, we made and compared WT, *swi5*, *ace2* and *swi5 ace2* strains, and took samples at 5 minute intervals for at least 40 minutes after release from the metaphase arrest. Hierarchical clustering analysis on the wt, *ace2*, *swi5 ace2* and *swi5* datasets provides a list of genes that are activated specifically by Ace2 and Swi5 or by both factors (Figure S1). Although a few genes seem to be very specific targets of just one of the two factors, most Swi5/Ace2 targets show at least some response to both factors. We found about 6 genes that are strongly and fairly specifically activated by Ace2: *SCW11, DSE1, DSE2, CTS1, SUN4,* and *GAT1*. Presumably these genes are expressed primarily in daughter cells. There are about 16 genes fairly specifically activated by Swi5: *EXG1, YPL066W, YMR122W-A, FAA3, PCL2, YBR071W, YOL019W, YPL088W, YLR194C, YAL053W, CRH1, MSC7, CHS1, SSO1, ACA1,* and *VCX1*. Finally there are at least 22 genes responding to a greater or lesser extent to both Ace2 and Swi5: *RME1, PST1, PIR1, ASH1, HIS1, SIC1, PCL9, YPL158C, CYK3, YLR049C, YNL046W, PRR1, YLR414C, FTH1, ESF2, ISR1, BUD9, PRY3, DSE3, DSE4, EGT2,* and *AMN1.* The classification of genes as "specific" to one or the other factors is somewhat arbitrary. In

addition, there are quite a number of genes that are weakly regulated (e.g., *CDC6*, apparently somewhat Swi5-specific). Note that a few genes are found at two locations in the cluster shown in Fig. S1 (e.g., *PIR1*, *FAA3*, *SUN4*); this is because the arrays contained two different probes (from two different regions of the transcription unit); each probe of different sequence is represented separately. Similar microarray data were also collected for an *ACE2** (i.e., localization defective) strain (not shown).

We obtained similar *ash1* vs. *ASH1** and *ace2 ash1* vs. *ACE2* ASH1** datasets, and performed similar statistical analysis, to obtain a list of genes whose expression is strongly regulated by Ash1 (p-value<10^{-3}). We found only two genes that display strong changes in expression upon deletion or mislocalization of Ash1: *HO* and *PST1*. Both genes, as expected, are repressed by Ash1. Analysis of the clusters obtained by subtracting the Ash1 deleted dataset from the Ash1 mislocalized dataset shows that the Swi5 and Ace2/Swi5 targets have higher expression in the absence of Ash1, indicating that Ash1 represses their expression (Figure S2). Ash1 may therefore be in general a modulator of Swi5-dependent expression, repressing the expression of many Swi5 and Swi5/Ace2 targets in daughter cells.

To obtain a list of shared Ace2 and Ash1 targets, we used the data obtained by doing a time point by time point subtraction of the gene deletion data from the mislocalized mutant data. The probability (p-value) that a gene was not affected by a given factor was computed via a χ^2 test. The error-bars for every time point were obtained by computing the standard deviation of the entire subtracted dataset. Assuming that the expression of the great majority of genes is not affected by Ace2 and Ash1, this quantity gives a good estimation of the measurement error of the microarrays. As typical for microarray data, our data were log-normally distributed (data not shown) and therefore all the analysis was performed using the $\log_2$ of the expression values.

We selected as acceptable Ace2/Ash1 targets only the genes that were detected as a differential signal in the subtracted arrays from all three datasets (*ace2* vs. *ACE2**, *ash1* vs. *ASH1** and *ace2 ash1* vs. *ACE2* ASH1**). We also imposed the condition that the observed changes in expression must be dependent on the localization of Ace2 and Ash1, by requiring that the Ace2/Ash1-dependent differences in expression should be manifested only after anaphase, when these factors accumulate in the daughter nucleus.

We imposed for both conditions a p-value of 0.05. For these values and given the size of the yeast genome (~6000), the number of expected false positive is 0.64, below one. This suggests that there is a high probability of getting zero or only one false positive. The p-values for the identified targets are reported in Table S3.

A direct interaction between Ace2 or Ash1 and the promoter of 3 of the identified targets (Ace2: *CLN3* and *HSP150*, Ash1: *YRF1-1*) has been previously observed in ChIP-chip experiments (Simon et al. (2001) Cell, *106*, 697-708, Harbison et al. (2004) Nature, *431*, 99-104), supporting the validity of our analysis. *YRF1* is a gene repeated 7 times in the yeast genome. While not perfectly conserved, the promoter regions of these 7 genes are very similar. The promoter region of *YRF1-5* is basically identical to that of *YRF1-1* (identical from -854 to +1), supporting its presence in our list. Ace2 has also been shown to bind to the *YRF1-2* promoter (Harbison et al. (2004) Nature, 431, 99-104).

Activation of the G1/S regulon in synchronized cell populations.

Ace2 and Ash1 regulate the expression of G1 cyclin *CLN3*. In particular, presence of Ace2 delays high expression of *CLN3* at M/G1 by about 5 minutes (see Figure 5b) while Ash1 represses *CLN3* expression in early G1 (see Figure 5c). As a consequence of this regulation, expression of the G1/S regulon should be delayed in the presence of Ace2 and Ash1. A lower and delayed activation of SBF/MBF genes in the presence of Ace2 and Ash1 was observed in the arrays of the single mutants (Figure S3a and S3b). Analysis of SBF/MBF activation in *ace2 ash1* and *ACE2* ASH1** shows that the G1/S regulon is activated with similar kinetics (Figure S3c). This is probably due to a difference in cell size at the time of birth after release from the *cdc20* arrest (Figures S3d and S3e). This difference implies that *ace2 ash1* cells activate the G1/S regulon at a smaller size than *ACE2* ASH1** as expected given the higher expression of *CLN3*.

Bioinformatics analysis to detect Ace2 and Swi5 regulatory sites.

To gain further insights on the regulation of *CLN3* expression by Ace2 and Swi5, we undertook a bioinformatics approach. We looked for regulatory motifs in the promoters of the identified Ace2 and Swi5 targets of 4 evolutionary related yeast strains (*S. cerevisiae*, *S. paradoxus*, *S. mikatae*, *S. bayanus*) using PhyloGibbs (Siddharthan et al.

(2005) PLoS Comput. Biol. 1:e67). Inclusion of phylogenetic information has been shown to improve the detection of regulatory motifs. This analysis provides a consensus site for Ace2 and Swi5 (GCTGG, Figure S11a) similar to the one previously reported (GCTGGT) (Harbison et al. (2004) Nature, 431, 99-104) and suggests possible binding sites for Ace2 and Swi5 in the promoters of their target genes. Two putative Ace2/Swi5 binding sites are found on the *CLN3* promoter (Figure S11b). These sites are perfectly conserved across the 4 species (GCTGG at -701 $p<10^{-12}$, GCTTGG at -569 $p<10^{-12}$). The first site coincides with one of the 5 Ace2/Swi5 putative sites we mutated. The second site is different from the consensus site and was not mutated. This site could maybe explain the residual binding to the *CLN3* promoter when all the consensus Ace2/Swi5 sites are mutated.
